# Descending Dysploidy and Bidirectional Changes in Genome Size Accompanied *Crepis* (Asteraceae) Evolution

**DOI:** 10.3390/genes12091436

**Published:** 2021-09-17

**Authors:** Magdalena Senderowicz, Teresa Nowak, Magdalena Rojek-Jelonek, Maciej Bisaga, Laszlo Papp, Hanna Weiss-Schneeweiss, Bozena Kolano

**Affiliations:** 1 Faculty of Natural Sciences, Institute of Biology, Biotechnology and Environmental Protection, University of Silesia in Katowice, 40-007 Katowice, Poland; senderowicz.magdalena@gmail.com (M.S.); teresa.nowak@us.edu.pl (T.N.); magdalena.rojek@us.edu.pl (M.R.-J.); maciej.bisaga@us.edu.pl (M.B.); 2 Eötvös Loránd University Botanical Garden, Illés u. 25, 1083 Budapest, Hungary; papplaca@gmail.com; 3 Department of Botany and Biodiversity Research, University of Vienna, Rennweg 14, A-1030 Vienna, Austria; hanna.schneeweiss@univie.ac.at

**Keywords:** chromosome number, karyotype formula, flow cytometry, genome size, phylogenetic analysis

## Abstract

The evolution of the karyotype and genome size was examined in species of *Crepis* sensu lato. The phylogenetic relationships, inferred from the plastid and nrITS DNA sequences, were used as a framework to infer the patterns of karyotype evolution. Five different base chromosome numbers (*x* = 3, 4, 5, 6, and 11) were observed. A phylogenetic analysis of the evolution of the chromosome numbers allowed the inference of *x* = 6 as the ancestral state and the descending dysploidy as the major direction of the chromosome base number evolution. The derived base chromosome numbers (*x* = 5, 4, and 3) were found to have originated independently and recurrently in the different lineages of the genus. A few independent events of increases in karyotype asymmetry were inferred to have accompanied the karyotype evolution in *Crepis*. The genome sizes of 33 *Crepis* species differed seven-fold and the ancestral genome size was reconstructed to be 1C = 3.44 pg. Both decreases and increases in the genome size were inferred to have occurred within and between the lineages. The data suggest that, in addition to dysploidy, the amplification/elimination of various repetitive DNAs was likely involved in the genome and taxa differentiation in the genus.

## 1. Introduction

Chromosomal changes, both numerical and structural, are acknowledged to be important mechanisms that accompany speciation and diversification in plants [1]. The large variation in chromosome numbers in the plant kingdom is the result of two major mechanisms—polyploidy and dysploidy [2]. Polyploidisation (whole genome duplication) and subsequent diploidisation seem to play a greater role in the evolution of angiosperms than in other eukaryotes [3]. There is evidence that most, if not all, angiosperms are ancient polyploids [4]. Dysploidy involves various types of structural rearrangements of chromosomes, which often result in decreases or increases in chromosome numbers [5,6]. The chromosome morphology can also be altered by the amplification/loss of DNA sequences [7]. Accumulation and/or loss of DNA sequences (mainly repetitive DNA) are mechanisms that lead to changes in the total karyotype length and genome size [8,9].

*Crepis* sensu lato (s.l.), which consists of approximately 200 annual and perennial species, is one of the largest genera in Asteraceae [10]. Most *Crepis* species are diploid with relatively low numbers of rather large and well-differentiated chromosomes. Several chromosome base numbers (*x* = 3, 4, 5, 6, and 11 [11]) and significant differences in genome size from 0.72 pg/1C to 32.75 pg/1C [12] have been reported in this genus previously. Thus, *Crepis* has been an excellent system for investigating the evolution of the karyotype [13,14]. The first cytogenetic and taxonomical study of numerous *Crepis* species defined several karyomorphotypes based on the number and morphology of the chromosomes and the symmetry/asymmetry of the karyotypes [15]. The first comprehensive sectional classification of *Crepis* was largely based on a combination of chromosomal and morphological characters of the species [11,15,16]. Thus, the genus *Crepis* has been considered to be the first model plant group in which chromosomal evolution plays an important role in speciation (e.g., [13,17,18,19]). The morphological, cytological and physiological evidence suggested that a decrease in the base chromosome numbers and a shortening of the life cycle accompanied the evolution of the genus [20]. Decreases in the chromosome numbers from an ancestral 2*n* = 12 to the derived 2*n* = 10, 8, and 6 have been suggested to have occurred mostly via the reciprocal translocations between non-homologous chromosomes [20]. In the diversification and speciation of the genus *Crepis*, the decreases in the base chromosome numbers and increases in karyotype asymmetry were suggested to be the main direction of the evolution of the karyotype [13,20]. 

Symmetrical karyotypes composed of mostly metacentric and submetacentric chromosomes of similar sizes were usually considered to be plesiomorphic. Asymmetrical karyotypes, considered to be apomorphic, are composed of highly variable in size chromosomes. Most of their chromosomes have terminally or subterminally localised centromeres [13,21]. This suggested that the most derived *Crepis* species possessed *x* = 3 chromosomes, asymmetrical karyotypes that were primarily annuals [13,20]. 

Molecular phylogenetic studies of the genus *Crepis* sensu lato (s.l.) tested Babcock’s hypothesis in a phylogenetic framework [22] and proposed a new classification of this genus. Three evolutionary lineages were distinguished based on the analyses of 75 taxa: (i) species with a chromosome base number *x* = 7, corresponding to Babcock’s section *Ixeridopsis* and now placed in genus *Askellia*; (ii) Babcock’s sections *Intybellia*, *Lagoseris*, *Phaecasium*, *Microcephalum*, and *Pterotheca*, as well as two other genera, *Lapsana* and *Rhagadiolus*, now included in the *Lagoseris* evolutionary lineage; and (iii) *Crepis* sensu stricto (*Crepis* s.s.), which comprised the remaining analysed *Crepis* species [22,23]. The new sections were highly heterogeneous compared to Babcock’s sections, also concerning base chromosome numbers, suggesting that the infrageneric classification of Babcock did not represent natural groups [20,22,23].

Modern cytogenetic methods combined with molecular phylogenetic analyses greatly facilitate the analyses of the trends in chromosomal evolution that accompany and/or follow diversification and speciation in plants. The aim of the study was to analyse the patterns of chromosome number and genome size evolution in *Crepis* s.l. species. Chromosome numbers, karyotype structure, genome size, and DNA sequence information were obtained de novo for *Crepis* species and *Lapsana communis* included in this study. All of the analyses were performed on the same set of species and individuals, which enabled the perfect correlation of the molecular and cytogenetic data.

## 2. Materials and Methods

### 2.1. Plant Material

Fifty-five accessions of 45 species of *Crepis* representing two evolutionary lineages *Lagoseris* and *Crepis* s.s. as well as *Lapsana communis*, which, according to Enke et al. [23], belongs to the *Lagoseris* evolutionary lineage, were analysed (Table 1). The analysed samples represented about a quarter of all currently recognized *Crepis* species [20]. *Picris hieracioides*, *Lactuca serriola*, and *Sonchus oleraceus* were used as outgroups for phylogenetic analyses. The plants were grown from seeds in the greenhouse of the University of Silesia in Katowice under a 16 h/8 h photoperiod at 19 ± 2 °C. The vouchers were deposited at the Herbarium KTU (University of Silesia, Chorzów, Poland; Table 1).

### 2.2. DNA Amplification and Sequencing

Total genomic DNA was isolated from fresh leaf tissue using a modified CTAB method [24]. Genomic DNA from each sample was checked for quality and quantity using an ND-1000 (peqLab, Erlangen, Germany). The PCR amplification of the nuclear rDNA ITS (internal transcribed spacer; nrITS) region was carried out according to Venora et al. [25] using the primers ITS4 and ITS5 [26]. Based on the analyses of Shaw et al. [27], the four non-coding plastid DNA spacer regions (*rpl*32*-trn*L, *rps*16*-trn*K, *ndh*C*-trn*V and *psb*D*-trn*T), which have a high number of potentially informative characters in euasterids II, were selected for the analyses. The cpDNA markers were amplified using the primers from Shaw et al. ([28]; Table S1). The PCR mixture for the amplification contained a 1x Color OptiTaq PCR Master Mix (EURx, Gdansk, Poland), 0.5 µM of each forward and reverse primer (Genomed, Warsaw, Poland), and 50 ng of the DNA template. The GeneAmpPCR System 9700 (Applied Biosystems, Waltham, MA, USA) and the conditions described in Blöch et al. [29] were used for the amplification. The PCR products were treated with *E. coli* Exonuclease I and FastAP Thermosensitive Alkaline Phosphatase (Thermo Fisher Scientific, Waltham, MA, USA) according to the manufacturer’s instructions and the cycle sequencing was performed using a 3730xl DNA Analyzer (Applied Biosystems; USA) in a commercial facility (Genomed; Warsaw, Poland or Macrogen; Seoul, Korea). The template DNAs that were used for the nrITS and cpDNA amplifications were always derived from the same isolate. All of the sequences were deposited in GenBank (accession numbers in Table 1).

### 2.3. Sequence Alignment and Phylogenetic Analyses

Most of the phylogenetic analyses were performed using the DNA sequences of four concatenated cpDNA regions and the nrITS that was obtained in this study. Additionally, an extended nrITS dataset, which contained the sequences that were obtained in this study as well as previously published sequences were also analysed [12]. The sequences were assembled using DNA Baser version 3 (Heracle BioSoft S.R.L., Pitesti, Romania). Multiple sequence alignments were performed 20 times using webPRANK [30] and MergeAlign [31] was then used to obtain a consensus of the multiple sequence alignments. The phylogenetic relationships were inferred independently for the nrITS and for the four concatenated plastid regions using the maximum likelihood, as implemented in IQ-TREE version 0.9.5 [32]. The best model of sequence evolution for the ML analyses was determined using the Bayesian information criterion as implemented in IQ-TREE [33]. The best fit models were TIM3e + G4 for nrITS and TVM + F + G4 for the plastid data sets. The significance of the inferred relationships was assessed via bootstrapping with 1000 replicates. *Picris hieracioides*, *Lactuca serriola*, and *Sonchus oleraceus* were used as the outgroup taxa. The resulting phylogenetic trees were created using FigTree v.1.3.1 [34]. Bootstrap support values below 75 were excluded from the figures.

### 2.4. Chromosome Preparation and Karyotype Analyses

Young leaves were pretreated with 2 mM 8-hydroxyquinoline (Sigma, Burlington, MA, USA) for 2 h at room temperature and for 2 h at 4 °C, fixed in methanol:glacial acetic (3:1) and stored at −20 °C until use. The mitotic metaphase chromosome spreads were prepared according to Dydak et al. [35]. The chromosome preparations were stained with DAPI (4′,6-diamidino-2-phenylindole) and analysed under a Zeiss AxioImager.Z.2 fluorescent microscope (ZEISS, Germany). The karyotype analyses were performed on at least ten high-quality mitotic metaphase spreads per accession. The chromosome length was measured using ImageJ software ver. 1.50 [36]. The chromosomes of each analysed cell were arranged into the karyotypes by decreasing length. The chromosome nomenclature of Levan et al. [37] was used. The degree of karyotype asymmetry was estimated according to Paszko [38]. The asymmetry index is an indicator of the levels of the heterogeneity of chromosome length and the positions of the centromeres in a given karyotype. The asymmetry index (A_1_) was calculated using the following equation:

(1) $$\mathrm{AI}\;=\;\frac{CV_{CL}\;\times \;CV_{CI}}{100}$$

where *CV_CL_* is the relative variation in chromosome length; *CV_CL_* = $\frac{S_{CL}}{X_{CL}}\;\times \;100$ (*S_CL_* is a standard deviation and *X_CL_* is the mean chromosome length), whereas *CV_CI_* is the relative variation centromeric index *CV_CI_* = $\frac{S_{CI}}{X_{CI}}\;\times \;100$ (*S_CI_* is the standard deviation and *X_CI_* is the mean centromeric index).

### 2.5. Genome Size Measurements

The genome sizes were measured using flow cytometry. *Brachypodium hybridum* Catalán, Joch.Müll., Hasterok & G. Jenkins ABR113 (2C DNA = 1.265 pg; [39]); *Solanum lycopersicum* L., Stupicke’ (1.96 pg/2C DNA; [40]; *Solanum pseudocapsicum* L. (2.58 pg/2C DNA, [41]); *Zea mays* L. ‘CE-777’ (2C = 5.43 pg/2C DNA, [42]); *Pisum sativum* L. ‘Ctirad’ (9.09 pg/2C DNA, [43]); and *Secale cereale* subsp. *cereale* L. (16.01 pg/2C DNA, [43]) were used as the internal standards, depending on the genome sizes of the measured samples. The youngest fully developed leaves of the analysed *Crepis* species and the appropriate internal standard (the information about the standards were added to Table 2 in results section) were chopped together in a Petri dish in 500 μL of a nuclei extraction buffer using a razor blade (CyStain PI Precision P Sysmex 05-5022). The nuclei suspension was filtered through a 30 μm mesh (CellTrics, Sysmex, Kobe, Japan) and stained with a staining buffer containing propidium iodide, RNase (CyStain PI Sysmex Precision P 05-5022, Kobe, Japan) and 1% β-mercaptoethanol (Sigma, Burlington, MA, USA) according to the manufacturer’s instructions. The samples were then analysed using a flow cytometer (CyFlow Space, Sysmex, Kobe, Japan) equipped with a 532 nm green laser. At least 10,000 nuclei were analysed for each sample. The sizes of the nuclear genomes were calculated as the linear relationship between the ratio of the 2C DNA peaks of a sample and the standard. Due to the high content of secondary metabolites, which prevented a flow cytometry analysis, the genome size could not be estimated for 12 species. The accepted CVs were less than 5%, except for *C. kotschyana* (5.78%) and diploid *C. vesicaria* (5.93%), for which measurements with CVs lower than 6% were accepted. The 1C*x*-value and DNA content of one non-replicated monoploid genome with chromosome number *x* were calculated according to Greilhuber et al. [44].

### 2.6. Ancestral State Reconstructions

The phylograms that resulted from the ML analysis (branch length information included) were used to infer the evolution of the chromosome numbers, the karyotype asymmetry, and genome size. The analyses were performed using the better supported cpDNA phylogram. Previously published genome size values for *Crepis* (Tables S2 and S3) [12] were added to the newly obtained results and together mapped on the phylogram resulting from the ML analysis of the extended nrITS dataset. The maximum likelihood analyses were performed under the CONST_RATE model (for the nrITS data set) or the CONST_RATE_DEMI model (for the cpDNA dataset), as implemented in ChromEvol 2.0. software [45]. For the ChromEvol analyses, the best-fit model was tested using an AIC test (Table S4). For the best-fitted model, the analyses were rerun with parameters that were fixed to those that were optimised in the first run using 10,000 simulations to compute the expected number of changes along each branch as well as the ancestral haploid chromosome numbers at the nodes. *Picris hieracioides*, *Sonchus oleraceus*, and *Lactuca serriola* were used to root the tree. Bootstrap support values less than 75 were excluded. The chromosome base numbers for *Crepis pontana*, *C. acuminata*, *C. atribarba*, *C. modocensis*, and *C. intermedia* were obtained from the literature [20,46] because of problems with obtaining good-quality meristematic tissue for the chromosomal analyses. The base chromosome number for the polyploid *C. biennis* (*x* = 5) was adopted from [47]. The evolution of the asymmetry index and genome sizes were analysed using maximum likelihood as implemented in the package phytools in the R software [48].

## 3. Results

### 3.1. Phylogenetic Analysis

The phylogenetic relationships of the 45 *Crepis* species and *Lapsana communis* were inferred from analyses of their nrITS sequences and four plastid regions (*rpl*32*-trn*L, *ndh*C*-trn*V, *rps*16*-trn*K and *trn*T*-psb*D). A total of 61 nrITS and 244 cpDNA sequences (61 sequences per each of the four chloroplast markers) were analysed. Three species, *Picris hieracioides*, *Lactuca serriola*, and *Sonchus oleraceus*, were used as the outgroups. The length of the isolated nrITS regions among the analysed species ranged from 540 to 598 bp. The final alignment was 668 bp long (including gaps) with 241 Characters that were parsimony informative. Only one ribotype of the nrITS was amplified for all of the studied accessions. The total length of the analysed concatenated plastid DNA regions ranged from 2948 to 3577 bp (the data for the individual chloroplast markers are listed in Table S5). The concatenated alignment of the cpDNA was 5693 bp long (including gaps) with 720 characters that were parsimony informative.

**Figure 1:**
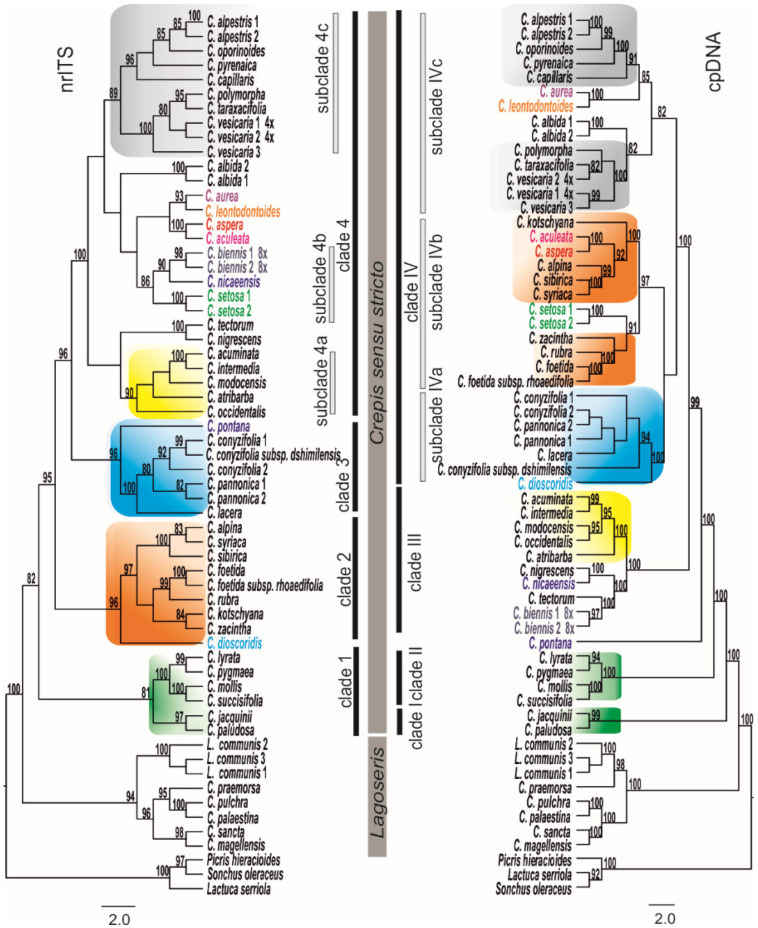
Phylogenetic relationships among the analysed *Crepis* species based on the nrITS and the cpDNA data sets. Bootstrap support values are indicated at each node. Names of the species that were recovered in different positions in the two datasets are indicated in colour. The trees were rooted with *Picris hieracioides*, *Lactuca serriola*, and *Sonchus oleraceus*.

Two major well-supported clades (nrITS BS100 and cpDNA BS100) corresponding to the evolutionary lineages of *Lagoseris* and *Crepis* s.s. (Figure 1) were consistently recovered in both the nrITS and cpDNA ML analyses. *Crepis* s.s., which contained most of the analysed species, was monophyletic in both of the nrITS and cpDNA analyses; however, the nrITS- and cpDNA-tree topologies differed in their branching patterns. The clades that were recovered in the cpDNA marker analyses were labelled with Roman numerals (clades I–IV) and the subclades with Roman numerals and lower-case letters (subclades IVa–IVc). The clades in the nrITS phylogenetic analyses were labelled with Arabic numerals (clades 1–4) and the subclades were labelled with Arabic numerals and lower-case letters (subclades 4a–4c). Four clades were distinguished in *Crepis* s.s. in all of the analyses. Clade 1 (BS81), which was recovered in the nrITS data analysis, included five perennial European species and the central Asian *C. lyrata*. This group of species was monophyletic in the nrITS phylogeny, but not in the cpDNA analysis in which the species were placed in two highly supported clades instead: clade I (BS99) and clade II (BS100; Figure 1). The second monophyletic clade 2 (BS96), which was recovered in the nrITS analysis, comprised nine, mostly annual *Crepis* species, with the exception of the perennial *C. sibirica*. These species were placed in subclade IVb (BS97) in the cpDNA tree together with *C. setosa* (Figure 1). Six European and/or Middle East perennial species, which were recovered in clade 3 (BS96) of the nrITS tree, were placed in subclade IVa (BS100) in the cpDNA phylogeny. Nearly half of the analysed *Crepis* s.s. species were placed in clade 4 in the nrITS phylogeny. Three highly supported subclades could be distinguished within this clade: (i) subclade 4a (BS90), which consisted of species that are native to North America; (ii) subclade 4b (BS86), which comprised C*. biennis*, *C. setosa*, and *C. nicaeensis*; and (iii) subclade 4c, which comprised both annual and perennial species from Europe and North Africa (from Algeria to Morocco). However, in the cpDNA phylogeny, the North American species together with *C. tectorum*, *C. nigrescens*, *C. nicaeensis*, and *C. biennis* formed the monophyletic clade III (BS100).

Three main evolutionary lineages were distinguished in the phylogenetic tree that resulted from the analysis of the newly obtained nrITS sequences and previously published data (species of *Askellia*. *Lagoseris* and *Crepis* s.s.; Figure S1; Table S3; [22]). Among the species of *Crepis* s.s., five main clades were identified, in congruence with earlier reports [22,23]. 

### 3.2. Chromosome Number

**Figure 2:**
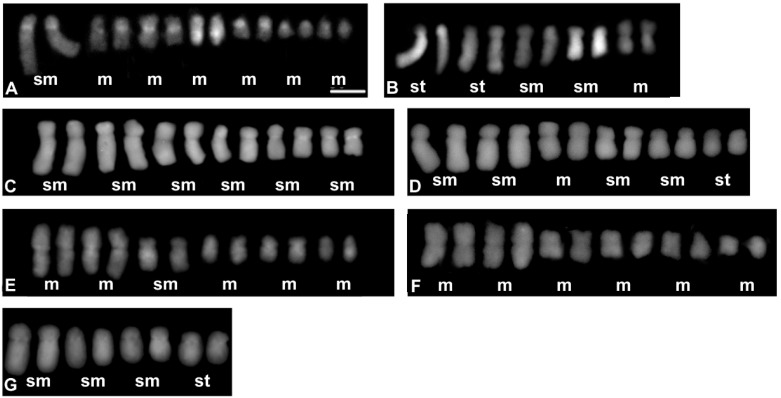
Karyograms representing each of the karyotype formula distinguished among the analysed *Crepis* species: (**A**) *Lapsana communis*; (**B**) *C. magellensis*; (**C**) *C. jacquinni*; (**D**) *C. paludosa*; (**E**) *C. succisifolia*; (**F**) *C. lyrate*; (**G**) *C. nicaeensis*. Letters below each pair of chromosomes indicate the type of chromosome: m—metacentric; sm—submetacentric; and st—subtelocentric. The metaphase plates used to prepare the karyograms are presented in Figure S2. Scale bar = 5 µm.

**Figure 3:**
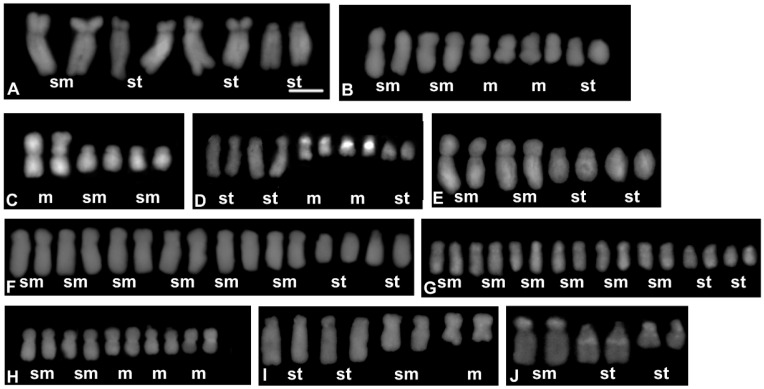
Karyograms representing each of the karyotype formula distinguished among the analysed *Crepis* species: (**A**) *C. pannonica*; (**B**) *C. foetida* subsp. *rhoaedifolia*; (**C**) *C. zacintha*; (**D**) *C. syriaca*; (**E**) *C. kotschyana*; (**F**) *C. vesicaria* 2; (**G**) *C. vesicaria* 1; (**H**) *C. leontodontoides*; (**I**) *C. oporinoides*; (**J**) *C. capillaris*. Letters below each pair of chromosomes indicate the type of chromosome: m—metacentric; sm—submetacentric; and st—subtelocentric. The metaphase plates used to prepare the karyograms are presented in Figure S3. Scale bar = 5 µm.

The chromosome numbers were analysed for 50 accessions (Table 2) representing 40 *Crepis* species and three accessions of *L. communis*. Most of the analysed *Crepis* species were diploids and only four polyploids were found, *C. biennis* (2*n* = 8*x* = 40), *C. occidentalis* (2*n* = 4*x* = 44), and *C. vesicaria*, which were represented by two polyploid accessions (both 2*n* = 4*x* = 16) and one diploid accession (2*n* = 2*x* = 8; Table 2; Figure S2). Five different chromosome base numbers were found among the diploid *Crepis* species: *x* = 3, 4, 5, 6, and 11 (Table 2; Figure 2, Figure 3 and Figure S4). The most common chromosome base numbers were *x* = 4 found in 20 species and *x* = 5 found in ten species. Six species had *x* = 6 and only two species had a chromosome base number of *x* = 3 (*C. capillaris* and *C. zacintha*). The chromosome base number of *x* = 11 was found in *C. occidentalis*, a representative of the American *Crepis* agamic complex (2*n* = 4*x* = 44; Figure S5). *L. communis* from the *Lagoseris* clade was diploid with 2*n* = 14. 

**Figure 4:**
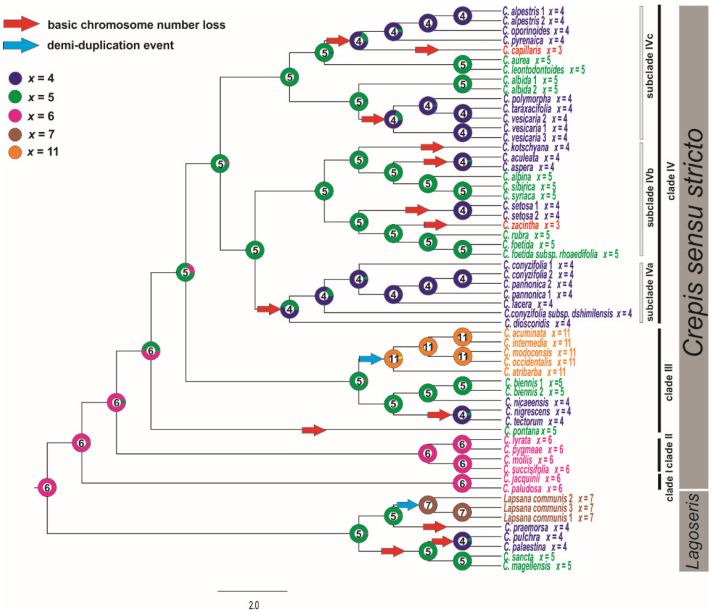
Ancestral character state reconstruction of the chromosome base numbers of the analysed species of *Crepis* s.l. The chromosome base numbers have been mapped on the ML tree of concatenated cpDNA sequences using the maximum likelihood method implemented in ChromEvol 2.0 software [45]. The tree was rooted with *Picris hieracioides*, *Lactuca serriola*, and *Sonchus oleraceus*.

The ML analysis (ChromEvol 2.0) was based on the cpDNA due to better support obtained for this marker. The ancestral chromosome base number for the common ancestor of *Lagoseris* and *Crepis* s.s. was inferred as *x* = 6 (pp = 0.97; Figure 4). The analysis of the cpDNA data sets suggested fourteen reductions (with expectation above 0.5) in the chromosome base numbers (Figure 4), three in the *Lagoseris* group and eleven in *Crepis* s.s. The ancestral base numbers for clades I and II was reconstructed as *x* = 6. The chromosome base number of *x* = 5 was inferred for the common ancestor of the species in clades III and IV (Figure 4). There was a further decrease in the chromosome base number from *x* = 5 to *x* = 4 in the evolutionary lineage of *C. tectorum* and *C. nigrescens* and in the evolutionary lineage of *C. nicaeensis* in clade III (Figure 4). A reduction in the chromosome base number from *x* = 5 to *x* = 4 accompanied the diversification of the common ancestor of the species in subclade IVa (Figure 4). Several events of descending dysploidy were also inferred for subclades IVb and IVc: (i) from *x* = 5 to *x* = 4 in the *C. kotschyana* lineage and *C. setosa* lineages; independently in the group that consisted of *C. taraxacifolia*, *C. vesicaria*, and *C. polymorpha*; in the lineages of *C. aculeata* and *C. aspera*; as well as for the common ancestor of the group, consisting of *C. alpestris*, *C. oporinoides*, *C. pyrenaica*, and *C. capillaris*; (ii) from *x* = 5 to *x* = 3 in *C. zacintha*; and (iii) from *x* = 4 to *x* = 3 in the *C. capillaris* lineage (Figure 4). 

### 3.3. Karyotype Structure

**Figure 5:**
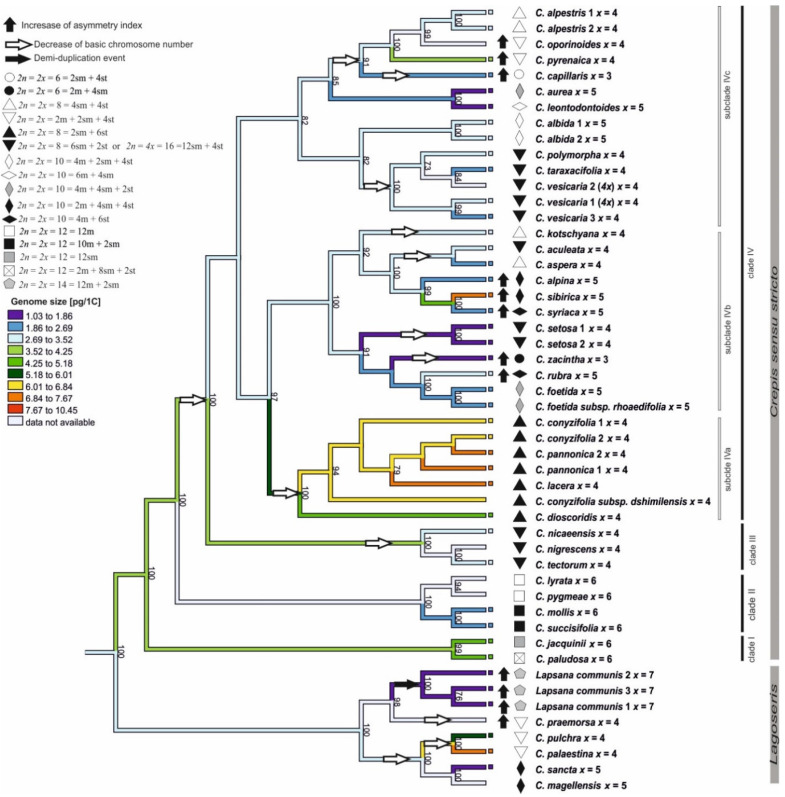
Karyotype evolution in the analysed species of *Crepis* s.l. Genome sizes were mapped on the ML tree of concatenated cpDNA sequences using the maximum likelihood method implemented in Phytools. Karyotype formulas (symbols preceding the species names) and the increases in karyotype asymmetry (black arrows) are indicated for all species. The genome sizes, karyotype formulas, and asymmetry indexes of each species are presented in Table 2. The figure includes only species for which all three datatypes (chromosome number, karyotype formula, and asymmetry index) were provided. The tree was rooted with *Picris hieracioides*, *Lactuca serriola*, and *Sonchus oleraceus*.

A morphometric analysis of the chromosomes was performed for 47 accessions representing 38 *Crepis* species and three accessions of *L. communis*. The majority of the analysed species (38 accessions of 31 species) had symmetrical karyotypes (Table 2). Only nine of the analysed *Crepis* species had karyotypes with a higher asymmetry index (Table 2). The asymmetry index (AI) values were mapped onto the ML phylogenetic tree using the maximum likelihood (Figure 5 and Figure S6). The analysis was performed using better supported the cpDNA phylogenetic trees. The symmetrical karyotype was inferred as the ancestral state for the common ancestor of *Lagoseris* and *Crepis* s.s. (Figure 5). In the evolutionary lineage of *Lagoseris*, an increase in the karyotype asymmetry accompanied the speciation of *C. praemorsa* and *L. communis*. An increase in the karyotype asymmetry accompanied also the evolution of several *Crepis* s.s. species from subclades IVb in the cpDNA tree (*C. alpina*, *C. sibirica*, *C. syriaca*, *C. rubra*, and *C. zacintha*) and from subclade IVc (*C. oporinoides*, *C. pyrenaica*, and *C. capillaris;* Figure 5). 

Fourteen different karyotype formulas were found based on the morphometric analyses of *Crepis* chromosomes (Table 2 and Figure 2, Figure 3 and Figure S4). Four different karyotype formulas were observed in the species from clade I and clade II that had the same chromosome base number as the ancestral recovered state (*x* = 6). The karyotypes of the species from clade II mainly consisted of metacentric chromosomes (*C. lyrata* and *C. pygmeae* with the karyotype formula 2*n* = 2*x* = 12 = 12m; Figure 2F and Figure S2; *C. succisifolia* and *C. mollis* with the karyotype formula 2*n* = 2*x* = 12 = 10m + 2sm; Figure 2E and Figure S4; Table 2), whereas the karyotypes of the species from clade I mainly had submetacentric chromosomes (*C. jacquinii* with 2*n* = 2*x* = 12 = 12sm; and *C. paludosa* with 2*n* = 2*x* = 12 = 2m + 8sm + 2st; Figure 2C,D; Table 2). 

Ten of the analysed species with a chromosome base number of *x* = 5 had five different karyotype formulas. The karyotypes of most of the species consisted of metacentric, submetacentric, and subtelocentric chromosomes (Figure 2, Figure 3 and Figure S4) and represented three different karyotype formulas: (i) 2*n* = 2*x* = 10 = 4m + 2sm + 4st (*C. albida*; Figure S4; Table 2); (ii) 2*n* = 2*x* = 10 = 2m + 4sm + 4st (*C. magellensis*, *C. sibirica*, *C. alpina* and *C. sancta*; Figure 2B and Figure S4; Table 2); and (iii) 2*n* = 2*x* = 10 = 4m + 4sm + 2st (*C. aurea* and *C. foetida*; Figure S4; Table 2). The karyotype of *C. leontodontoides* exclusively consisted of metacentric and submetacentric chromosomes (2*n* = 2*x* = 10 = 6m + 4sm; Figure 3H). Two other species (*C. syriaca* and *C. rubra*; Figure 3D and Figure S4; Table 2) had metacentric and subtelocentric chromosomes (2*n* = 2*x* = 10 = 4m + 6st). The species with *x* = 5 were mainly included in subclades IVb and IVc; however, even closely related species with the same chromosome number differed in their karyotype formulas (e.g., *C. aurea* and *C. leontodontoides*; Figure 5). Among the species with a chromosome base number of *x* = 4, four different karyotype formulas were distinguished. Species from clade III and the group of species that is closely related to *C. vesicaria* (from subclade IVc) had karyotypes with submetacentric and subtelocentric chromosomes (2*n* = 2*x* = 8 = 6sm + 2st or 2*n* = 4*x* = 8 = 6sm + 2st; Figure 3G,H and Figure S4; Table 2). This type of karyotype formula was recovered for *C. aculeata* and *C. setosa* (subclade IVb; Figure S4; Table 2). In subclade IVa, all of the species had the karyotype formula of 2*n* = 2*x* = 8 = 2sm + 6st (Figure 5 and Figure 3A; Table 2; Figure S4). Species with the karyotype formula 2*n* = 2*x* = 8 = 4sm + 4st were present in subclades IVb (*C. kotschyana*, *C. aspera*) and IVc (*C. alpestris*; Figure 5 and Figure S4; Table 2). Five species with a chromosome base number of *x* = 4 had karyotypes with metacentric, submetacentric, and subtelocentric chromosomes. The karyotype formula 2*n* = 2*x* = 2m + 2sm + 4st was present in two species from subclade IVc (*C. oporinoides* and *C. pyrenaica*) and three species from the *Lagoseris* evolutionary lineage (*C. palaestina*, *C. pulchra*, and *C. praemorsa*; Figure 5 and Figure S4; Table 2). Two species with a chromosome base number *x* = 3 belonged to two different subclades (Figure 5) and had two different karyotype formulas (2*n* = 2*x* = 6 = 2m + 4sm for *C. zacintha*; Figure 3C; Table 2; and 12*n* = 2*x* = 6 = 2sm + 4st for *C. capillaris*; Figure 3J; Table 2).

### 3.4. Genome Size

The genome sizes (1C values) of the 33 *Crepis* species and for *L. communis* were measured (Table 2). The genome sizes varied nearly seven-fold among the diploid species, ranging from 1.03 pg/1C DNA (*C. zacintha*) to 7.46 pg/1C DNA (*C. lacera*). The genome sizes of the species of the *Lagoseris* evolutionary lineage were variable with nearly a six-fold difference (1.22 pg/1C DNA to 7.05 pg/1C DNA). In *Crepis* s.s., larger genome sizes were found in two species from clade I (mean 1C value = 4.78 pg), whereas clade II species had smaller genome sizes (mean 1C value = 2.44 pg). In clade III, two diploid species *C. tectorum* (3.06 pg/1C DNA) and *C. nicaeensis* (3.17 pg/1C DNA) had small genomes, whereas the octoploid *C. biennis* had a larger genome (10.45 pg/1C DNA). The C*x*-value (the C-value of monoploid genome “*x*”) of *C. biennis* was rather small (1C_x_ = 2.61 pg/1C DNA; Figure 5 and Figure S7) compared to the diploid species of this group. Three different groups of related species that differed in the patterns of their genome sizes distribution were identified in clade IV. The subclade IVa consisted of diploid species with larger genome sizes (mean 1C value = 6.35 pg) and, among them, *C. lacera* had the largest estimated genome size (1C value = 7.46 pg). The genome sizes of the species from clade IVc were on average 2.52 pg/1C DNA. The largest variation in genome sizes was observed among the species in subclade IVb in which the genome size of *C. sibirica* (1C value = 6.98 pg) was nearly seven times larger than that of *C. zacintha* (1C value = 1.03 pg; Table 2; Figure 5 and Figure S7). *C. vesicaria* contained both diploid and tetraploid accessions (2*n* = 8 and 2*n* = 16). The genome sizes that were estimated for the diploid and tetraploid accessions (2.43 pg/1C and 2.78 pg/1C, respectively). 

The DNA amounts were mapped onto the cpDNA ML phylogenetic tree using the maximum likelihood method (Figure 5 and Figure S7). A genome size of 3.2 pg/1C DNA was inferred as the ancestral state for the *Lagoseris* lineage. Both decreases and increases in genome sizes were inferred to have accompanied speciation and diversification of this clade (Figure 5 and Figure S7). The ancestral genome size for the entire *Crepis* s.s. lineage was reconstructed as 3.70 pg/1C. For clade I, 4.8 pg/1C was reconstructed as the ancestral state, whereas 2.50 pg/1C was recovered as the ancestral genome size for clade II. For the entire clade IV, 3.2 pg/1C was reconstructed as the ancestral genome size. For subclade IVa, the ancestral genome size was reconstructed to be 5.5 pg/1C, whereas for subclades IVb and IVc, 2.7 pg/1C and 2.9 pg/1C, respectively, were recovered. An increase in genome size was inferred for the species of clade I and the octoploid *C. biennis* (clade III). In contrast, a reduction in genome size accompanied the evolution of many species of clade II and subclades IVb and IVc (e.g., *C. aurea* and *C. zacintha*). The evolution of a common ancestor of subclade IVa was accompanied by an increase in the genome size, with further increases or decreases in the genome sizes inferred during the diversification and speciation of this clade.

The inferences of genome size evolution were additionally performed using nrITS tree based on an extended data set containing both newly obtained genome size data and previously reported genome size estimations (Figure S8; Table S2). The extended data set included the species from the American agamic complex, which had relatively large genome sizes (Table S3). The observed values of the reconstructed ancestral states were slightly larger in comparison to the results of the analyses of the newly obtained data only. The inferred events of increases and reductions in genome size were very similar in both data sets. 

## 4. Discussion

Resolving the phylogenetic relationships for *Crepis* provided the evolutionary framework for elucidating the evolution of the chromosome numbers and karyotype structure. Phylogeny based on the biparentally inherited nrITS of mostly diploid species of *Crepis* was found to be largely congruent with previously published results (Figure S1; [22,23]). In the present study, a new clade comprising species of the North American *Crepis* agamic complex was identified. These species have not been included in previous analyses. The phylogenetic relationships inferred in this study were inconsistent with the sections defined by Babcock [20], in agreement with the earlier phylogenetic analyses [22]. Each clade and subclade contained species previously classified in different Babcock sections (Figure S1). A phylogenetic analysis, based on the chloroplast markers, recovered mostly the same main groups of species as the nrITS-based analyses. However, the relationships among these clades were found to be incongruent between the nuclear and plastid markers. Such nucleo-cytoplasmic discordance has been well documented in previous studies of higher plants [49]. This incongruence could be explained by the convergent evolution of shared chloroplast sequences, incomplete lineage sorting of the ancestral polymorphisms, or introgressive hybridization [50,51]. The *Crepis* species analysed here were mostly diploids, but because *Crepis* species are primarily cross-pollinated and many of them hybridise extensively [20], introgression could have accompanied the speciation of individual species, as also suggested for other plant groups [52,53]. The incongruence could be also explained by technical reasons. Several nodes in nrITS phylogram in clade 4 were weakly supported and the analysed species represent only a quarter of the *Crepis* genome, which may influence the results. The incongruence between the nrITS and cpDNA trees concerning the position of *C. biennis* could suggest its allopolyploid origin. *C. biennis* is an octoploid species [47] whose origin and parental species are not yet known. The present analysis suggests a putative ancestral species for the two subgenomes of this polyploid taxon, species that are similar to the extant *C. nicaeensis/C. setosa* and another species related to *C. tectorum* (Figure 1). Additional molecular phylogenetic and cytogenetic studies are necessary to elucidate the origin of *C. biennis*.

This study reports the genome sizes for 33 species of *Crepis* s.l., eight of them for the first time. The genome sizes that were reported in the current study are largely in agreement with previous reports, except for two species: *C. vesicaria* and *C. pannonica* (Table 2; [12,54,55]). This discrepancy is probably the result of methodological factors as the previous data were obtained using Feulgen densitometry or flow cytometry but with different standards. In the case of the diploid *C. vesicaria*, the discrepancy (2.43 pg/1C in this study versus 4.18 reported by Enke et al. [12]) could result from the presence of the diploid and polyploid cytotypes within this species [20]. A strong correlation between genome size and life form has been suggested for *Crepis* in previous studies [12]. The current results, however, did not show an obvious correlation between these two characters. Both annual and perennial *Crepis* species groups showed high levels of variation of their genome sizes. Both one of the smallest (*C. leontodontoides* 1.06 pg/1C) and the largest genomes (*C. lacera*; 7.46 pg/1C) were found in the perennial diploid species. The correlation between genome size and plant life form has been extensively discussed in the literature. The annual life form, especially in weedy species, was inferred to be associated with a smaller genome size compared to its related perennials [56,57,58]. Such a correlation, however, must be interpreted with caution because other ecological and evolutionary factors might also influence genome size evolution [59]. The chromosome numbers of *Crepis* species analysed in the current study were in accordance with earlier reports [20,60]. Most of the analysed species were diploids, with only a few polyploids [20]. The tetraploids of *C. vesicaria* shared the same karyotype structure with its diploid accession, which suggests that they were of autopolyploid origin

Analyses of chromosome number and genome size evolution in a phylogenetic framework revealed that there was no general trend in their evolution among angiosperms [61,62,63]. Both chromosome numbers and genome sizes could change very rapidly during the evolution and diversification of plant genomes [64,65,66]. A reduction in genome size was earlier suggested to accompany the evolution of the genus *Crepis* [12,67], with increases in genome sizes mainly correlated with polyploidisation [20]. However, the present data revealed a bidirectional evolution of genome size in both the *Lagoseris* and *Crepis* s.s. evolutionary lineages. The current study revealed that several diploid species have a genome size that is much larger than the ancestral reconstructed states of the lineages they belong to, e.g., *C. pannonica* or *C. pulchra*. This suggested a bidirectional evolution of genome size in at least some of the *Crepis* lineages. The proliferation of repetitive DNA sequences is considered to be the major cause of the increase in genome size next to polyploidy [7,24,68]. The mechanisms of the reduction of genome sizes and DNA removal are less understood, with an unequal homologous recombination or illegitimate recombination proposed to be most important [69,70]. The information about the genome structure in *Crepis* is still very limited and only further analyses, especially of repetitive DNA fraction, will allow better insight into the mechanisms of the evolution of genome size in this genus. 

The ancestral states of the base chromosome number in a common ancestor of *Lagoseris* and *Crepis* s.s. was inferred to be *x* = 6, and descending disploidy was inferred as a main trend in the chromosome number evolution in *Crepis* (Figure 4). The obtained results are partly in agreement with the hypothesis of Babcock [20], who suggested that a decrease in the chromosome base number is the main trend in *Crepis* evolution. However, the derived base chromosome numbers (*x* = 5, 4, and 3) were found to have evolved independently several times in the evolutionary history of the genus. The chromosome base number of *x* = 5 has evolved at least three times during *Crepis* evolution. Dysploid change from *x* = 5 to *x* = 4 has occurred twice in *Lagoseris* and eight times in *Crepis* s.s. The base chromosome *x* = 3 has evolved twice in *Crepis* s.s. Nearly half of the descending dysploidy events were recovered for the same branches for which changes in genome sizes were inferred (Figure 5). Some descending dysploidy events were accompanied by a reduction in genome sizes whereas others were accompanied by genome size increases and no common trend was observed. Similar patterns have been reported for *Artemisia* (Asteraceae; [64]) and other plant genera (e.g., *Genlisea*, Lentibulariaceae; [65]). 

The chromosome base number was inferred to have increased only twice during the evolution of the analysed species. An allopolyploidisation or a duplication of the chromosome number followed by dysploidy is likely to have given rise to the base chromosome of *x* = 11 present in the American agamic complex. The evolution of this lineage was accompanied by an increase in genome size (Figure S8), supporting the hypothesis of the polyploidy origin of this new base chromosome number of *x* = 11. In contrast, the increase in base chromosome number in the evolutionary lineage of *Lapsana* (from *x* = 6 to *x* = 7) was accompanied by the genome size downsizing. The chromEvol analyses inferred that the changes in chromosome number in the *Lapsana* lineage resulted from demiduplication. For demiduplication, a genome increase instead of decrease would be expected. Unfortunately, the direct ancestor of *Lapsana* and its genome size are unknown and thus this hypothesis cannot be tested. More detailed comparative analyses of the karyotype structure of *Lapsana* and related taxa are necessary to gain insight into the origin of base chromosome number of *x* = 7 in the *Lagoseris* lineage. Changes in the chromosome base numbers as well as in genome sizes in *Crepis* were most prevalent at the tips of the tree rather than early in the evolution of the genus. Similar trends have been reported for *Trifolium*, *Hypochaeris*, or *Braschyscome* [71,72,73].

Chromosomal rearrangements often accompany plant diversification and speciation [74,75,76]. Chromosome rearrangements generally have a low level of homoplasy. Therefore, it was hypothesised that species with the same chromosome number that evolved independently should differ in their chromosome structure [1]. Molecular cytogenetic methods and the availability of many chromosomal markers enables the comparative analysis of the karyotype in model or cultivated taxa (e.g., Brassicaceae and *Brachypodium* [77,78,79]. In wild species groups it is more challenging, but even comparative analyses of the morphology of chromosomes are indicative of occurrences of chromosomal rearrangements. However, the shape and size of the chromosomes can also be altered by a sequence amplification/loss [1,7,8,9]. A comparative analysis of the *Crepis* karyotype formulas revealed that species with the same chromosome base number belonging to different evolutionary lineages differed in their karyotype morphologies (Figure 5). Moreover, even the species in the same subclades (e.g., subclade IVc) possessing the same chromosome base number differed in karyotype structure (Figure 5). These results supported the hypothesis that the derived base chromosome numbers (*x* = 5, 4, 3) might have evolved independently multiple times in *Crepis*. The changes in karyotype structure might lead to an increase in its asymmetry [38]. An increase in karyotype asymmetry was previously suggested as the main pattern of karyotype evolution in the genus *Crepis* [80]. The results of the present study did not support this hypothesis. An increase in karyotype asymmetry accompanied the speciation of only a few species in the different evolutionary lineages (Figure 5). The increases in karyotype asymmetry accompanied both decreases and an increase in the chromosome base numbers (Figure 5), as well as increases or decreases in the genome sizes. Some groups of closely related *Crepis* species had the same chromosome number and a similar karyotype structure (e.g., subclade IVa), which suggests that different mechanisms, e.g., an amplification of repetitive sequences, might have accompanied the diversification of these species.

## 5. Conclusions

Analyses of karyotype structure and genome sizes in a phylogenetic context enabled inferences about the ancestral base chromosome numbers and genome sizes in the *Lagoseris* and *Crepis* s.s. evolutionary lineages. A few events of base chromosome number changes, mostly descending dysploidy, explained the chromosome number distribution in the extant *Crepis* s.l. species best. Most of these changes accompanied the evolution of individual species or a small group of closely related, derived species. Analyses of the genome size evolution revealed both increases and decreases during the evolution of the genus. The changes in genome sizes occurred mainly at the tips rather than at the internal nodes of the tree. The present work contributes to a better understanding of the evolution of the genomes of *Crepis* and lays the foundation for more detailed comparative analyses of its karyotype structure and evolution, including repetitive sequences.

## Figures and Tables

**Table 1:** Species names, collection numbers, places of seed collecting, voucher numbers of the analysed taxa, and the GenBank accessions numbers of the sequences obtained in this study.

Taxon	Collection Details	Voucher	GenBank Accession Number				
			*trn*K*-rps*16	*rpl*32*-trn*L	*trn*T*-psb*D	*ndh*C*-trn*V	nrITS
***Crepis* s.s.**							
*Crepis aculeata* Boiss.	BGT 38	KTU154623	MT234677	MT234860	MT234738	MT234799	MN549102
*C. acuminata* Nutt.	USDA W6 40086 N 41°20′31.217″ W 119°50′57.293″	-	MT234678	MT234861	MT234739	MT234800	MN549103
*C. albida* (1) Vill.	UGA 233	-	MT234679	MT234862	MT234740	MT234801	MN549111
*C. albida* (2) Vill.	JBI 2	KTU157715	MT234680	MT234863	MT234741	MT234802	MN549112
*C. alpestris* (1) (Jacq.) Tausch	BGUG N49	KTU157712	MT234681	MT234864	MT234742	MT234803	MN549104
*C. alpestris* (2) (Jacq.) Tausch	BGBD 708 N 47°46′43.18″ E 13°26′01.16″	KTU157714	MT234682	MT234865	MT234743	MT234804	MN549105
*C. alpina* L.	USDA PI 274367	KTU154609	MT234683	MT234866	MT234744	MT234805	MN549106
*C. aspera* L.	LBG 006722	KTU157716	MT234685	MT234868	MT234746	MT234807	MN549108
*C. atribarba* A.Heller	USDA W6 36843	-	MT234686	MT234869	MT234747	MT234808	MN549109
*C. aurea* (L.) Cass.	USDA PI 312843	KTU157719	MT234687	MT234870	MT234748	MT234809	MN549110
*C. biennis* L. 1	BGBD 656 N 51°11′16.44″ E 10°3′4572″	KTU154629	MT234688	MT234871	MT234749	MT234810	MN549113
*C. biennis* L. 2	Wołosate, Poland N 49°03′29.60″ E 22°42′08.92″	KTU157728	MT234689	MT234872	MT234750	MT234811	MN549114
*C. capillaris* Wallr.	BGGU 335	KTU154610	MT234691	MT234874	MT234752	MT234813	MN549116
*C. conyzifolia* (Gouan) A.Kern.	GBA 462	KTU157720	MT234692	MT234875	MT234753	MT234814	MN549117
*C. conyzifolia* (Gouan) A.Kern.	UGA 236	-	MT234693	MT234876	MT234754	MT234815	MN549118
*C. conyzifolia* subsp. *dshimilensis* (K.Koch) Lamond	GBBG	-	MT234714	MT234897	MT234775	MT234836	MN549132
*C. dioscoridis* L.	IPK CRE2	KTU154619	MT234694	MT234877	MT234755	MT234816	MN549119
*C. foetida* L.	USDA PI 296071	KTU154612	MT234695	MT234878	MT234756	MT234817	MN549120
*C. foetida* subsp. *rhoaedifolia* (M.Bieb.) Celak.	HBBH 1734	KTU154614	MT234718	MT234901	MT234779	MT234840	MN549137
*C. intermedia* A.Gray	USDA W6 52751 N 39°48′15.984″ W 119°56′2.867″	-	MT234696	MT234879	MT234757	MT234818	MN549121
*C. jacquinii* Tausch	Sarnia Skała Tatra Mts, Poland N 49°15′52.77″ E 19°56′30.36″	KTU159736	MT234697	MT234880	MT234758	MT234819	MT234671
*C. kotschyana* Boiss.	USDA PI 310392	KTU164608	MT234698	MT234881	MT234759	MT234820	MN549122
*C. lacera* Ten.	BGMN	KTU159735	MT234699	MT234882	MT234760	MT234821	MT234672
*C. leontodontoides* All.	BGDG 658 N 42°8′43.044″ E 8°59′32.964″	KTU154631	MT234700	MT234883	MT234761	MT234822	MN549123
*C. lyrata* (L.) Froel.	SSBG	-	MT234701	MT234884	MT234762	MT234823	MT234673
*C. modocensis* Greene	USDA W6 49189 N 45°3′19.368″ W 109°10′4.079″	-	MT234703	MT234886	MT234764	MT234825	MN549124
*C. mollis* Asch.	Sławków, Poland N 50°17′45.90″ E 19°16′59.06″	KTU154630	MT234704	MT234887	MT234765	MT234826	MN549125
*C. nicaeensis* Balb.	BGEU	KTU157730	MT234705	MT234888	MT234766	MT234827	MT234675
*C. nigrescens* Pohle	HUM	MW0553775	MT234706	MT234889	MT234767	MT234828	MN549126
*C. occidentalis* Nutt.	USDA W6 45275 N 43°19′50.7″ W 117°11′0.708″	-	MT234707	MT234890	MT234768	MT234829	MN549127
*C. oporinoides* Boiss. ex Froel.	ABGL 1516	KTU154622	MT234684	MT234867	MT234745	MT234806	MN549107
*C. paludosa* Moench	Sławków, Poland N 50°18′07.51″ E 19°21′19.10″	KTU154625	MT234709	MT234892	MT234770	MT234831	MN549128
*C. pannonica* (Jacq.) K.Koch 1	BGBD 256-01-00-14 N 48°21′58.54″ E 16°25′06.13″	KTU154627	MT234710	MT234893	MT234771	MT234832	MN549130
*C. pannonica* (Jacq.) K.Koch 2	BGEU	KTU157729	MT234711	MT234894	MT234772	MT234833	MT234676
*C. polymorpha* Pourr	JBN 149	KTU157725	MT234713	MT234896	MT234774	MT234835	MN549131
*C. pontana* Dalla Torre	ABGL 235	KTU154624	MT234690	MT234873	MT234751	MT234812	MN549115
*C. pygmaea* L.	UGA 239	KTU157722	MT234716	MT234899	MT234777	MT234838	MN549135
*C. pyrenaica* (L.) Greuter	BGBD 1010 N 42°52′22.44″ W 0°26′56.616″	KTU154621	MT234717	MT234900	MT234778	MT234839	MN549136
*C. rubra* L.	BGK 364	KTU154607	MT234719	MT234902	MT234780	MT234841	MN549138
*C. setosa* Haller f. 1	HBUR 1275	KTU154620	MT234722	MT234905	MT234783	MT234844	MN549140
*C. setosa* Haller f. 2	IPK CRE20	KTU157713	MT234721	MT234904	MT234782	MT234843	MN549141
*C. sibirica* L.	BGBD 738 N 60°21′34.37″ E 59°11′22.58″	KTU157721	MT234723	MT234906	MT234784	MT234845	MN549142
*C. succisifolia* Tausch	Rędziny, Poland N 50°49′08.66″ E 15°55′55.27″	KTU154656	MT234724	MT234907	MT234785	MT234846	MN549143
*C. syriaca* (Bornm.) Babc. & Navashin	KEW 0129064	KTU154615	MT234725	MT234908	MT234786	MT234847	MN549144
*C. taraxacifolia* Thuill.	BGGU 347	KTU157723	MT234726	MT234909	MT234787	MT234848	MN549145
*C. tectorum* L.	Ustroń, Poland N 49°43′14.68″ E 18°49′29.11″	KTU157717	MT234727	MT234910	MT234788	MT234849	MN549146
*C. vesicaria* L. 3	OBUP	KTU157724	MT234730	MT234913	MT234791	MT234852	MN549149
*C. vesicaria* L. 2	BGBD 918 N 39°34′24.816″ E 2°38′42.648″	KTU157726	MT234729	MT234912	MT234790	MT234851	MN549148
*C. veiscaria* L. 1	BGBD 1014	KTU154616	MT234728	MT234911	MT234789	MT234850	MN549147
*C. zacintha* (L.) Loisel.	BGT 92	KTU154606	MT234731	MT234914	MT234792	MT234853	MN549150
***Lagoseris***							
*C. magellensis* F.Conti & Uzunov	BGMN	KTU157727	MT234702	MT234885	MT234763	MT234824	MT234674
*C. palaestina* Bornm.	BGGU 335	KTU154611	MT234708	MT234891	MT234769	MT234830	MN549129
*C. pulchra* L.	BGGU 341	KTU154648	MT234712	MT234895	MT234773	MT234834	MN549134
*C. preamorsa* (L.) Tausch	BGBD 662 N 60°18′21.29″ E 10°35′20.76″	KTU154628	MT234715	MT234898	MT234776	MT234837	MN549133
*C. sancta* (L.) Bornm.	BGUK 104	KTU154613	MT234720	MT234903	MT234781	MT234842	MN549139
*Lapsana communis* L. 1	KEW 0018568	KTU154617	MT234733	MT234916	MT234794	MT234855	MN549151
*L. communis* L. 2	Rogoźnik Poland N 50°24′03.43″; E 19°01′59.96″	KTU157708	MT234734	MT234917	MT234795	MT234856	MN549152
*L. communis* L. 3	Rogoźnik Poland N 50°23′50.22″; E 19°01′48.50″	KTU157709	MT234735	MT234918	MT234796	MT234857	MN549153
***Outgroup***							
*Lactuca serriola* L.	Strzyżowice Poland N 50°23′33.47″ E 19°04′03.14″	KTU157718	MT234732	MT234915	MT234793	MT234854	MN549156
*Picris hieracioides* L.	Jaworzno Poland N 50°13′31.43″ E 19°16′28.63″	KTU157710	MT234736	MT234919	MT234797	MT234858	MN549154
*Sonchus oleraceus* L.	Ustroń, Poland N 49°42′58.97″ E 18°47′53.44″	KTU157711	MT234737	MT234920	MT234798	MT234859	MN549157

Voucher deposited in KTU; seed origin and accession number: (BGT) Botanic Garden of Tel Aviv University; (USDA) USDA North Central Regional Plant Introduction Station of the US National Plant Germplasm System; (UGA) Université Grenoble Alpes; (JBI) Jardín Botánico de Iturraran Lorategi Botanikoa, Spain; (BGUG) Botanical Garden of Universität Graz; (BGBD) Botanical Garden Freie Universität Berlin—Dahlem; (LBG) Lyon Botanical Garden, France; WB) Wołosate, Bieszczady National Park, Poland; (BGGU) The Botanical Garden of Göttingen University; (GBA) Giardino Botanico Alpino “Rezia”, Italy; (GBBG) Gruzja Batumi Botanical Garden; (IPK) The Leibniz Institute of Plant Genetics and Crop Plant Research (IPK), Germany; (HBBH) Hortus Botanicus Budapest, Hungary; BGMN) Botanical Gardens of Majella National Park, Italy; (SSBG) The South-Siberian Botanical Garden of Altai State University; (BGEU) Botanical Garden of Eötvös University in Budapest; (HUM) Herbarium Universitatis Mosquensis; (ABGL) Alpine Botanical Garden of Lautaret, France; (JBN) Jardin Botanique de Nancy; (BGK) Botanical Garden in Kiel; (HBUR) Hortus Botanicus Universitatis, Jassy, Romania; (KEW) Millenium Seed Bank KEW Gardens; (OBUP) Orto Botanico Dell Universito Di Padora Italia; (BGUK) Botanischer Garten Universität Konstanz, Germany.

**Table 2:** Species names, karyotype formulas, asymmetry indices, and genome size of the analysed taxa.

Species	Karyotype Formula *	Asymmetry Index (AI)	Genome Size pg/1C ± SD	Internal Standard
***Crepis* s.s.**				
*Crepis aculeata* Boiss.	2*n* = 2*x* = 8 = 6sm + 2st	1.38	2.89 ± 0.04	*Pisum sativum*
*C. albida* Vill. (1)	2*n* = 2*x* = 10 = 4m + 2sm + 4st	7.07	3.08 ± 0.03	*P. sativum*
*C. albida* Vill. (2)	2*n* = 2*x* = 10 = 4m + 2sm + 4st	7.39	-	-
*C. alpestris* (Jacq.) Tausch (1)	2*n* = 2*x* = 8 = 4sm + 4st	3.96	2.99 ± 0.03	*P. sativum*
*C. alpestris* (Jacq.) Tausch (2)	2*n* = 2*x* = 8 = 4sm + 4st	2.52	-	-
*C. alpina* L.	2*n* = 2*x* = 10 = 2m + 4sm + 4st	13.47	2.20 ± 0.05	*Zea mays*
*C. aspera* L.	2*n* = 2*x* = 8 = 4sm + 4st	4.24	2.15 ± 0.02	*Lycopersicon pseudocapsicum*
*C. aurea* (L.) Cass.	2*n* = 2*x* = 10 = 4m + 4sm + 2st	5.93	1.63 ± 0.10	*Lycopersicon esculentum*
*C. biennis* L. (2)	2*n* = 8*x* = 40	-	10.45 ± 0.33	*P. sativum*
*C. capillaris* Wallr.	2*n* = 2*x* = 6 = 2sm + 4st	20.15	2.07 ± 0.08	*Z. mays*
*C. conyzifolia* (Gouan) A.Kern. (1)	2*n* = 2*x* = 8 = 2sm + 6st	3.74	6.08 ± 0.16	*P. sativum*
*C. conyzifolia* (Gouan) A.Kern. (2)	2*n* = 2*x* = 8 = 2sm + 6st	2.06	-	-
*C. conyzifolia* subsp. *dshimilensis* (K.Koch) Lamond	2*n* = 2*x* = 8 = 2sm + 6st	3.67	-	-
*C. dioscoridis* L.	2*n* = 2*x* = 8 = 2sm + 6st	4.74	4.58 ± 0.13	*Secale cereale*
*C. foetida* L.	2*n* = 2*x* = 10 = 4m + 4sm + 2st	11.17	2.03 ± 0.08	*L. pseudocapsicum*
*C. foetida* subsp. *rhoaedifolia* (M.Bieb.) Celak.	2*n* = 2*x* = 10 = 4m + 4sm + 2st	10.92	2.17 ± 0.02	*L. pseudocapsicum*
*C. jacquinii* Tausch	2*n* = 2*x* = 12 = 12sm	5.93	5.12 ± 0.05	*S. cereale*
*C. kotschyana* Boiss.	2*n* = 2*x* = 8 = 4sm + 4st	5.54	2.92 ± 0.08	*P. sativum*
*C. lacera* Ten.	2*n* = 2*x* = 8 = 2sm + 6st	5.54	7.46 ± 0.10	*P. sativum*
*C. leontodontoides* All.	2*n* = 2*x* = 10 = 6m + 4sm	5.37	1.06 ± 0.03	*Brachypodium hybridum*
*C. lyrata* (L.) Froel.	2*n* = 2*x* = 12 = 12m	5.72	-	-
*C. mollis* Asch.	2*n* = 2*x* = 12 = 10m + 2sm	9.57	2.53 ± 0.04	*L. pseudocapsicum*
*C. nicaeensis* Balb.	2*n* = 2*x* = 8 = 6sm + 2st	3.74	3.17 ± 0.02	*P. sativum*
*C. nigrescens* Pohle	2*n* = 2*x* = 8 = 6sm + 2st	3.45	-	-
*C. oporinoides* Boiss. ex Froel.	2*n* = 2*x* = 8 = 2m + 2sm + 4st	16.56	-	-
*C. paludosa* Moench	2*n* = 2*x* = 12 = 2m + 8sm +2st	10.92	4.53 ± 0.19	*S. cereale*
*C. pannonica* (Jacq.) K.Koch (1)	2*n* = 2*x* = 8 = 2sm + 6st	6.52	7.27 ± 0.06	*P. sativum*
*C. pannonica* (Jacq.) K.Koch (2)	2*n* = 2*x* = 8 = 2sm + 6st	6.41	-	-
*C. polymorpha* Pourr	2*n* = 2*x* = 8 = 6sm +2st	1.61	3.13 ± 0.02	*P. sativum*
*C. pygmeae* L.	2*n* = 2*x* = 12 = 12m	7.53	-	-
*C. pyrenaica* (L.) Greuter	2*n* = 2*x* = 8 = 2m + 2sm + 4st	11.66	3.54 ± 0.05	*S. cereale*
*C. rubra* L.	2*n* = 2*x* = 10 = 4m + 6st	12.85	2.86 ± 0.07	*P. sativum*
*C. setosa* Haller f. (1)	2*n* = 2*x* = 8 = 6sm + 2st	10.66	1.67 ± 0.02	*L. esculentum*
*C. setosa* Haller f. (2)	2*n* = 2*x* = 8 = 6sm + 2st	7.30	-	-
*C. sibirica* L.	2*n* = 2*x* = 10 = 2m + 4sm + 4st	14.44	6.98 ± 0.04	*P. sativum*
*C. succisifolia* Tausch	2*n* = 2*x* = 12 = 10m + 2sm	10.77	2.34 ± 0.05	*L. pseudocapsicum*
*C. syriaca* (Bornm.) Babc. & Navashin	2*n* = 2*x* = 10 = 4m + 6st	75.39	2.39 ± 0.28	*L. pseudocapsicum*
*C. taraxacifolia* Thuill.	2*n* = 2*x* = 8 = 6sm + 2st	1.01	2.47 ± 0.58	*P. sativum*
*C. tectorum* L.	2*n* = 2*x* = 8 = 6sm + 2st	2.308	3.06 ± 0.05	*P. sativum*
*C. vesicaria* L. (3)	2*n* = 2*x* = 8 = 6sm + 2st	2.38	2.43 ± 0.03	*L. pseudocapsicum*
*C. vesicaria* L. (1)	2*n* = 4*x* = 16 = 12sm + 4st	2.68	2.78 ± 0.09	*P. sativum*
*C. vesicaria* L. (2)	2*n* = 4*x* = 16 = 12sm + 4st	3.05	-	-
*C. zacintha* (L.) Loisel.	2*n* = 2*x* = 6 = 2m+ 4sm	18.61	1.03 ± 0.02	*Brachypodium hybridum*
***Lagoseris***				
*C. magellensis* F.Conti & Uzunov	2*n* = 2*x* = 10 = 2m + 4sm + 4st	5.26	-	-
*C. palaestina* Bornm.	2*n* = 2*x* = 8 = 2m + 2sm + 4st	10.45	7.05 ± 0.19	*P. sativum*
*C. praemorsa* (L.) Tausch	2*n* = 2*x* = 8 = 2m + 2sm + 4st	27.15	-	-
*C. pulchra* L.	2*n* = 2*x* = 8 = 2m + 2sm + 4st	10.2	5.59 ± 0.12	*P. sativum*
*C. sancta* (L.) Bornm.	2*n* = 2*x* = 10 = 2m + 4sm + 4st	5.37	1.60 ± 0.02	*Z. mays*
*Lapsana communis* L. (1)	2*n* = 2*x* = 14 = 12 m + 2sm	12.06	1.22 ± 0.04	*Z. mays*
*L. communis* L. (2)	2*n* = 2*x* = 14 = 12 m + 2sm	12.70	-	-
*L. communis* L. (3)	2*n* = 2*x* = 14 = 12 m + 2sm	13.67	-	-

* m—metacentric chromosome, sm—submetacentric chromosome; st—subtelocentric chromosome.

## Data Availability

All data generated or analysed during this study are available from the corresponding author on reasonable request.

## Supplementary Materials

The following are available online at https://www.mdpi.com/article/10.3390/genes12091436/s1, Figure S1: Maximum likelihood phylogenetic tree based on nrITS sequences incorporating of taxa analysed in the present study and available in GenBank accessions of other species from *Crepis* s.l. and related taxa. All the species analysed in this study are indicated in bold. Bootstrap support values are indicated at each node, Figure S2: Metaphase plates of *Crepis* species used to prepare the karyograms in Figure 2 (A) *Lapsana communis*, (B) *C. magellensis*, (C) *C. jacquinni*, (D) *C. paludosa*, (E) *C. succisifolia*, (F) *C. lyrata*, (G) *C. nicaeensis*. Scale bar = 5 µm, Figure S3: Metaphase plates of *Crepis* species used to prepare the karyograms in Figure 3: (A) *C. pannonica*, (B) *C. foetida* subsp. *rhoaedifolia*, (C) *C. zacintha*, (D) *C. syriaca*, (E) *C. kotschyana*, (F) *C. vesicaria* 2, (G) *C. vesicaria* 1, (H) *C. leontodontoides*, (I) *C. oporinoides*, (J) *C. capillaris*. Scale bar = 5 µm, Figure S4: Metaphase chromosomes of analysed *Crepis* species: (A) *C. sancta*, (B) *C. palaestina*, (C) *C. pulchra*, (D) *C. praemorsa*, (E) *C. mollis*, (F) *C. pygmae*, (G) *C. tectorum*, (H) *C. nigrescens*, (I) *C. dioscoridis*, (J) *C. conyzifolia dshimilensis*, (K) *C. lacera*, (L) *C. conyzifolia*, (M) *C. foetida*, (N) *C. rubra*, (O) *C. setosa*, (P) *C. sibirica*, (Q) *C. alpina*, (R) *C. aspera*, (S) *C. aculeata*, (T) *C. vesicaria* 3, (U) *C. taraxacifolia*, (V) *C. polymorpha*, (W) *C. albida*, (X) *C. aurea*, (Y) *C. pyrenaica*, (Z) *C. alpestris*. Descriptions below each pair of chromosomes indicate the type of chromosome: m—metacentric; sm—submetacentric; and st—subtelocentric. The metaphase plates used to prepare karyograms are presented in Figure S9. Scale bar = 5 µm, Figure S5: Metaphase plate of *Crepis occidentalis* (A) and *C. biennis* (B), Figure S6: Ancestral character state reconstruction of the asymmetry indexes of karyotypes of analysed species of *Crepis* s.l. The asymmetry indexes have been mapped on the ML tree of concatenated cpDNA sequences using maximum likelihood method as implemented in phytools. The tree was rooted with *Picris hieracioides*, *Lactuca serriola*, and *Sonchus oleraceus*, Figure S7: Ancestral character state reconstruction of the genome size (1C) of species of *Crepis* s.l. The 1C DNA amounts have been mapped on the ML tree of cpDNA sequences using maximum likelihood reconstruction as implemented in phytools. Numbers next to the nodes indicate reconstructed ancestral 1C-values (in pg). The genome size of each species is presented in Table 2. The tree was rooted with *Picris hieracioides*, *Lactuca serriola*, and *Sonchus oleraceus*, Figure S8: Ancestral character state reconstruction of genome size (1C-value) of *Crepis* s.l. taxa analysed in the present study and available in literature. The analysis was conducted using maximum likelihood reconstruction as implemented in phytools, Figure S9: Metaphase plates of *Crepis* species used to prepare the karyograms in Figure S4: (A) *C. sancta*, (B) *C. palaestina*, (C) *C. pulchra*, (D) *C. praemorsa*, (E) *C. mollis*, (F) *C. pygmae*, (G) *C. tectorum*, (H) *C. nigrescens*, (I) *C. dioscoridis*, (J) *C. conyzifolia dshimilensis*, (K) *C. lacera*, (L) *C. conyzifolia*, (M) *C. foetida*, (N) *C. rubra*, (O) *C. setosa*, (P) *C. sibirica*, (Q) *C. alpina*, (R) *C. aspera*, (S) *C. aculeata*, (T) *C. vesicaria* 3, (U) *C. taraxacifolia*, (V) *C. polymorpha*, (W) *C. albida*, (X) *C. aurea*, (Y) *C. pyrenaica*, (Z) *C. alpestris*. Scale bar = 5 µm, Table S1: Sequences of primers used for PCR amplification and sequencing, Table S2: Species name and GenBank accessions numbers of the selected *Crepis* sequences from Enke et al. [22] used in this study, Table S3: Genome size of *Crepis* species analysed in present study and data retrieved from literature, Table S4: The ΔAIC scores and Akaike weights of each model tested in ChromEvol 2.0 software for nrITS and cpDNA data sets, Table S5: Length of analysed plastid DNA and nrITS (nuclear ITS: ITS1-5.8S rDNA-ITS2) data sets [81,82].
