# Supplementary material for: Descending Dysploidy and Bidirectional Changes in Genome Size Accompanied Crepis (Asteraceae) Evolution
Source: Genes (Basel). 2021 Sep 17;12(9):1436. doi: 10.3390/genes12091436 (PMC8472258; doi:10.3390/genes12091436)
Supplement: Supplementary file 1 [file genes-12-01436-s001.zip › Senderowicz_et_al_Table S1.pdf]

Table S1. Sequences of primers used for PCR amplification and sequencing. (White et al., 1990 ; Shaw et al., 2007)

Region	Primer name and sequence (5'-3')
ITS1-5.8S rDNA-ITS2	**ITS4**: TCC TCC GCT TAT TGA TAT GC<br>**ITS5**: GGA AGT AAA AGT CGT AAC AAG G
*3'trn*V-*ndh*C	***trn*V(UAC)x2**: GTC TAC GGT TCG ART CCG TA<br>***ndh*C**: TAT TAT TAG AAA TGY CCA RAA AAT ATC ATA TTC
*psb*D-*trn*T	***psb*D**: CTC CGT ARC CAG TCA TCC ATA<br>***trn*T**(GGU)-R: CCC TTT TAA CTC AGT GGT AG
3'*rps*16-5'*trn*K	***rps*16*x2F2***: AAA GTG GGT TTT TAT GAT CC<br>***trn*K$^{(UUU)}$*x1***: TTA AAA GCC GAG TAC TCT ACC
*rpl*32-*trn*L	***trn*L$^{(UAG):}$** CTG CTT CCT AAG AGC AGC GT<br>***rpl*32-*F***: CAG TTC CAA AA A AAC GTA CTT C
