# Supplementary material for: Descending Dysploidy and Bidirectional Changes in Genome Size Accompanied Crepis (Asteraceae) Evolution
Source: Genes (Basel). 2021 Sep 17;12(9):1436. doi: 10.3390/genes12091436 (PMC8472258; doi:10.3390/genes12091436)
Supplement: Supplementary file 1 [file genes-12-01436-s001.zip › Senderowicz_et_al_Table S2.pdf]

Table S2. Species name and GenBank accessions numbers of the selected *Crepis* sequences from Enke et al. 2008 used in this study

Species name	GenBank accession number
C. albida subsp. albida	EU363606
C. albida subsp. grosii	EU363594
C. albida subsp. scorzoneroides	EU363595
C. alpestris	AJ633373
C. alpina	EU363649
C. aspera	EU363628
C. aurea subsp. aurea	EU363627
C. auriculifolia	EU363626
C. baldaccii	EU363625
C. bellidifolia	EU363615
C. biennis	AJ633355
C. bungei	AJ633374
C. bursifolia	EU363623
C. capillaris	AJ633381
C. chondrilloides	EU363593
C. chrysantha subsp. chrysantha	EU363622
C. cretica	EU363614
C. crocea	EU363590
C. cytherea	EU363646
C. darvazica	EU363600
C. dioritica	EU363620
C. flexuosa	EU363596
C. foetida	EU363619
C. foetida subsp. rhoeadifolia	EU363613
C. frigida	EU363612
C. guioliana	EU363618
C. heldreichiana	EU363603
C. hierosolymitana	EU363602
C. hookeriana	EU363605
C. hypochaeridea	EU363617
C. jacquinii subsp. kerneri	EU363636
C. jaquinii	AJ633378
C. kotschyana	EU363635
C. lampsanoides	EU363599
C. leontodontoides	EU363592
C. macropus	EU363589
C. merxmuelleri	EU363644
C. mollis	AJ633380
C. multicaulis subsp. congesta	EU363642
C. nana	EU363591
C. neglecta subsp. corymbosa	EU363611
C. neglecta subsp. neglecta	EU363610
C. nigrescens	EU363609

C. oporinoides	EU363633
C. oreades	EU363640
C. palaestina	EU363639
C. paludosa	EU366428
C. pontana	AJ633275
C. praemorsa	EU363654
C. pulchra	AJ633369
C. purpurea	EU363653
C. pusilla	EU363650
C. pyrenaica	EU363624
C. rhaetica	AJ633379
C. rubra	AJ633350
C. sahendi	EU363651
C. sancta subsp. nemauensis	EU363632
C. setosa	EU363585
C. sibthorpiana	EU363648
C. smyrnaea	EU363598
C. sonchifolia	EU363637
C. thomsonii	EU363647
C. tingitana	EU363586
C. triasii	EU363597
C. turcomanica	EU363652
C. tybakiensis	EU363631
C. vesicaria subsp. haenseleri	AJ633371
C. vesicaria subsp. stellata	EU363630
C. viscidula subsp. geracioides	EU363629
C. zacintha	EU363655
C.foetida subsp. afghanistanica	EU363604
Hedypnois rhagadioloides	AJ633307
Heteracia szovitsii	AJ633283
Hololeion maximowiczii	AJ633425
Hyoseris radiata	AJ633299
Hypochoeris maculata	AJ633311
Ixeris chinensis	EU363587
Ixeris laevigata	EU363588
Ixeris stolonifera	AJ633284
Lapsana communis	AJ633285
Phitosia crocifolia	EU363621
Picris echioides	AJ633321
Prenanthes purpurea	AJ633342
Rhagadiolus edulis	AJ633297
Rhagadiolus stellatus	AJ633296
Sonchus oleraceus	AY862581
Soroseris glomerata	EU363656
Taraxacum bessarabicum	ZJ633287
Taraxacum erythrospermum	AJ633291
Taraxacum laevigatum	AJ633288
Youngia denticulata	AJ633293

Youngia japonica	AJ633294
Youngia tenuifolia	EU363645
