# Supplementary material for: Descending Dysploidy and Bidirectional Changes in Genome Size Accompanied Crepis (Asteraceae) Evolution
Source: Genes (Basel). 2021 Sep 17;12(9):1436. doi: 10.3390/genes12091436 (PMC8472258; doi:10.3390/genes12091436)
Supplement: Supplementary file 1 [file genes-12-01436-s001.zip › Senderowicz_et_al_Table S3.pdf]

Table S3. Genome size of *Crepis* species analysed in present study and data retrieved from literature

Species	Genome size (pg/1C ± SD present study	Genome size (pg/1C ± SD) data taken from literature
Crepis aculeata	2.89 ± 0.04	-
C. acuminata (2*x*)		13.8 ± 0.4 [1]
C. albida	3.08 ± 0.03	-
C. alpestris	2.99 ± 0.03	-
C. alpina	2.20 ± 0.05	3.00 [2]
C. aspera	2.15 ± 0.02	-
C. atribarba (2*x*)		13.3 ± 0.7 [1]
C. aurea	1.63 ± 0.10	1.75 ± 0.07 [3]
C. biennis	10.45 ± 0.33	9.51 ± 0.492 [3]
C. bungei		4.05 ± 0.053 [3]
C. capillaris	2.07 ± 0.08	-
C. chrysantha subsp. chrysantha		4.64 ± 0.053 [3]
C. conyzifolia	6.08 ± 0.16	-
C. crocea (4*x*)		9.61 ± 0.053 [3]
C. dioscoridis	4.58 ± 0.13	-
C. foetida	2.03 ± 0.08	1.46 ± 0.034 [3]
C. foetida subsp. rhoaedifolia	2.17 ± 0.02	-
C. jacquinni	5.12 ± 0.05	-
C. kotschyana	2.92 ± 0.08	-
C. lacera	7.46 ± 0.10	-
C. lapsanoides		5.6 [4]
C. leontodontoides	1.06 ± 0.03	1.14 ± 0.012 [3]
C. mollis	2.53 ± 0.04	-
C. modocensis (2*x*)		12.2 ± 0.3 [1]
C. multicaulis subsp. congesta		1.78 ± 0.018 [3]
C. nicaeensis	3.17 ± 0.02	-
C. occidentalis (4*x*)		23.8 ± 1.7 [1]
C. palaestina	7.05 ± 0.19	6.10 [4]
C. paludosa	4.53 ± 0.19	4.53 ± 0.074 [3]
C. pannonica	7.27 ± 0.06	-
C. polymorpha	3.13 ± 0.02	-
C. pontana		6.9 [4]
C. pulchra	5.59 ± 0.12	5.50 [5]
C. pusilla		1.11 ± 0.025 [3]
C. praemorsa		5.3 [4]
C. pyrenaica	3.54 ± 0.05	3.58 ± 0.051 [3]
C. rubra	2.86 ± 0.07	2.90 [4]

C. sancta	1.60 ± 0.02	-
C. setosa	1.67 ± 0.02	1.70 [5]
C. sibirica	6.98 ± 0.04	-
C. succisifolia	2.34 ± 0.05	-
C. syriaca	2.39 ± 0.28	-
C. taraxacifolia	2.47 ± 0.58	-
C. tectorum	3.06 ± 0.05	-
C. vesicaria (1) 4x	2.78 ± 0.09	-
C. vesicaria (3) 2x	2.43 ± 0.03	-
C. viscidula subsp. gieracioides		4.90 [5]
C. zacintha	1.03 ± 0.02	-
Lapsana communis	1.22 ± 0.04	-

References

[1] Sears, C.J., Whitton, J., 2016. A reexamination of the North American Crepis agamic complex and comparison with the findings of Babcock and Stebbins' classic biosystematic monograph. American Journal of Botany 103, 1289-1299.

[2] Wallace, H., Sparkes, C.A., Maden, M., 1972. Nuclear DNA content of three Crepis species. Heredity 29, 367-373

[3] Enke, N, Fuchs, J, Gemeinholzer, B. 2011. Shrinking genomes? Evidence from genome size variation in Crepis (Compositae). Plant Biology 13: 185-193.

[4] Bennett, MD, Smith, JB. 1976. Nuclear-DNA Amounts in Angiosperms. *Philosophical Transactions of the Royal Society B-Biological Sciences* **274:** 227-274.

[5] Dimitrova, D, Greilhuber, J. 2000. Karyotype and DNA-content evolution in ten species of Crepis (Asteraceae) distributed in Bulgaria. *Botanical Journal of the Linnean Society* **132:** 281-297.
