# Supplementary material for: Descending Dysploidy and Bidirectional Changes in Genome Size Accompanied Crepis (Asteraceae) Evolution
Source: Genes (Basel). 2021 Sep 17;12(9):1436. doi: 10.3390/genes12091436 (PMC8472258; doi:10.3390/genes12091436)
Supplement: Supplementary file 1 [file genes-12-01436-s001.zip › Senderowicz_et_al_Table S4.pdf]

Table S4. The ΔAIC scores and Akaike weights of each model tested in ChromEvol 2.0 software for nrITS and cpDNA data sets.

Model*	Dataset			
	ITS		cpDNA	
	ΔAIC	Akaike weight	ΔAIC	Akaike weight
CR	**0**	**0.45**	0.7	0.34
CRD	0.2	0.40	**0**	**0.48**
CRDE	2	0.16	2	0.18
CRND	18.4	0.00	25.6	0.00
LR	108.3	0.00	101.4	0.00
LRD	108.3	0.00	101.7	0.00
LRDE	131.4	0.00	126.3	0.00
LRND	228	0.00	101.1	0.00

*CR – const_rate, CRD – const_rate_demi, CRDE – const_rate_demi_est, CRND – const_rate_no_dupl, LR – linear_rate, LRD – linear_rate_demi, LRDE – linear_rate_demi_est, LRND – linear_rate_no_dupl,
