# Supplementary material for: Descending Dysploidy and Bidirectional Changes in Genome Size Accompanied Crepis (Asteraceae) Evolution
Source: Genes (Basel). 2021 Sep 17;12(9):1436. doi: 10.3390/genes12091436 (PMC8472258; doi:10.3390/genes12091436)
Supplement: Supplementary file 1 [file genes-12-01436-s001.zip › Senderowicz_et_al_Table S5.pdf]

Table S5. Length of analyzed plastid DNA and nrITS (nuclear ITS: ITS1-5.8S rDNA-ITS2) data sets.

Data set	sequence length		
*rpl*32-*trn*L	*rpl*32 gene<br>18 bp	*rpl32-trn*L spacer<br>436 - 874 bp	-
*rps*16-*trn*K	-	*rps*16-*trn*K spacer<br>664 – 729 bp	-
*psb*D-*trn*T	-	*trn*T-*psb*D spacer<br>760 – 1076 bp	-
*ndh*C-*trn*V	-	*ndh*C-*trn*V spacer<br>734 – 988 bp	-
nrITS	ITS1<br>167 - 180 bp	5,8S rDNA<br>158 bp	ITS2<br>201 – 262 bp
