# Supplementary figures and images for: Descending Dysploidy and Bidirectional Changes in Genome Size Accompanied Crepis (Asteraceae) Evolution
Source: Genes (Basel). 2021 Sep 17;12(9):1436. doi: 10.3390/genes12091436 (PMC8472258; doi:10.3390/genes12091436)

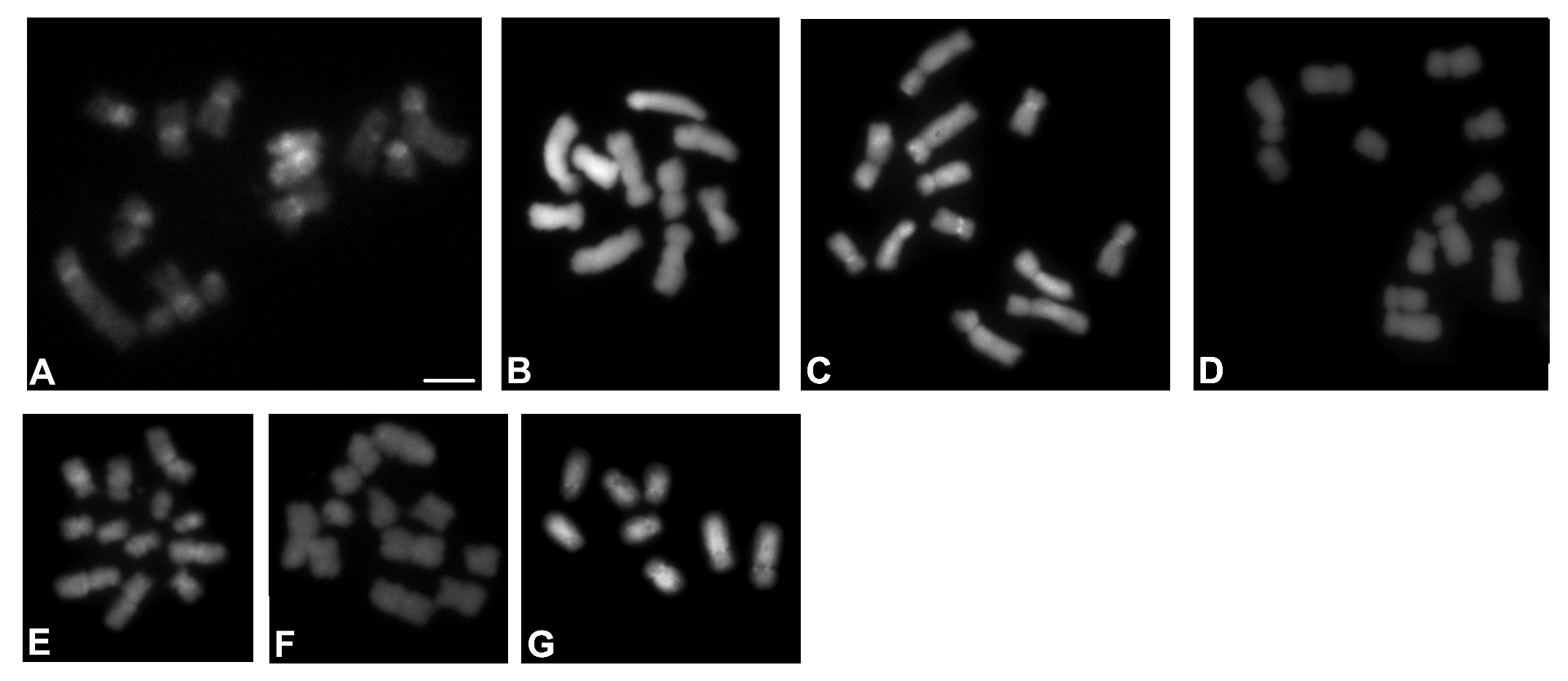

Supplement: Supplementary file 1 [file genes-12-01436-s001.zip › Senderowicz_et_al_Figure S2.tif]

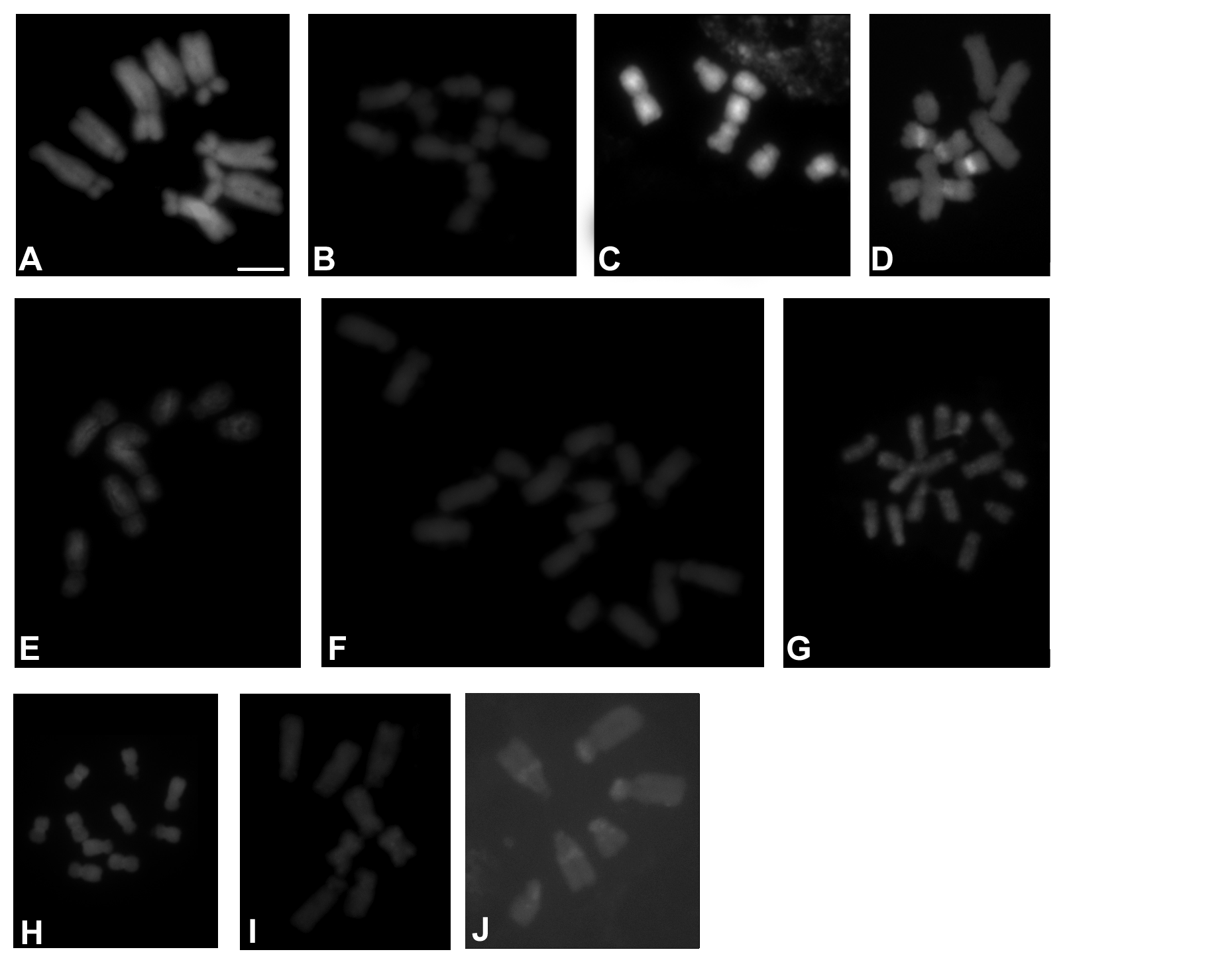

Supplement: Supplementary file 1 [file genes-12-01436-s001.zip › Senderowicz_et_al_Figure S3.tif]

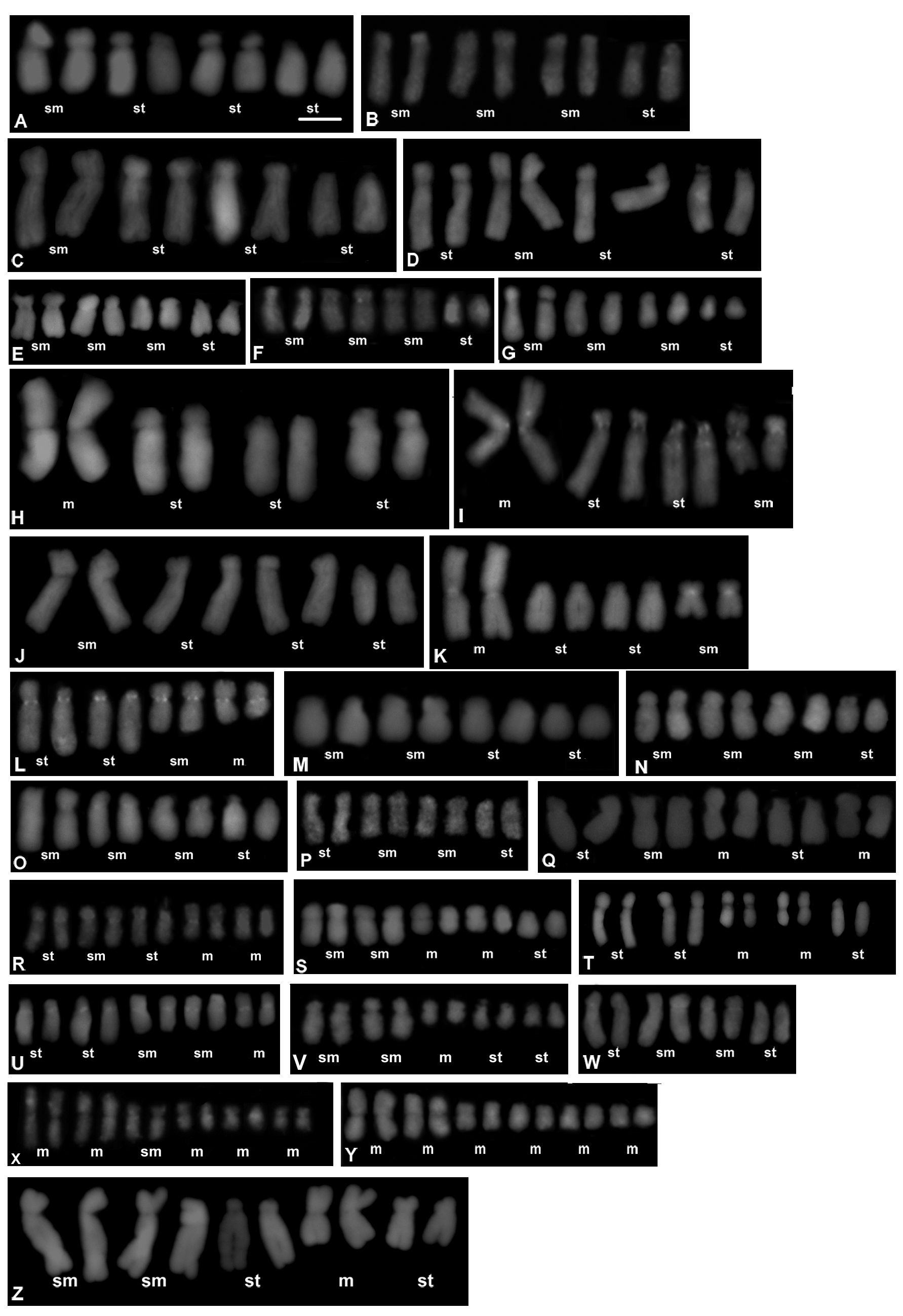

Supplement: Supplementary file 1 [file genes-12-01436-s001.zip › Senderowicz_et_al_Figure S4.tif]

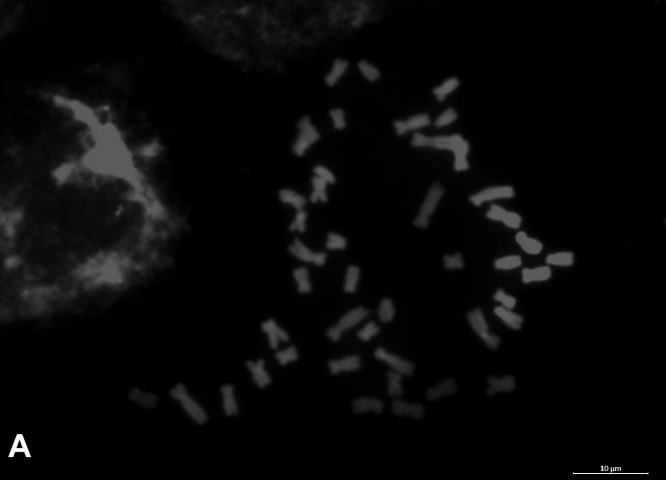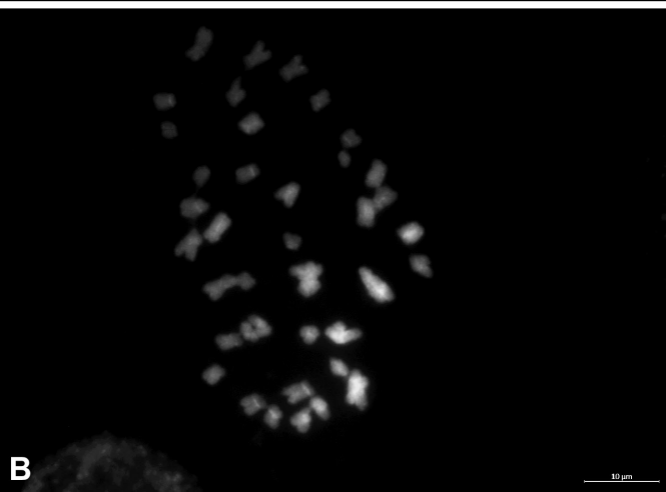

Supplement: Supplementary file 1 [file genes-12-01436-s001.zip › Senderowicz_et_al_Figure S5.pdf]

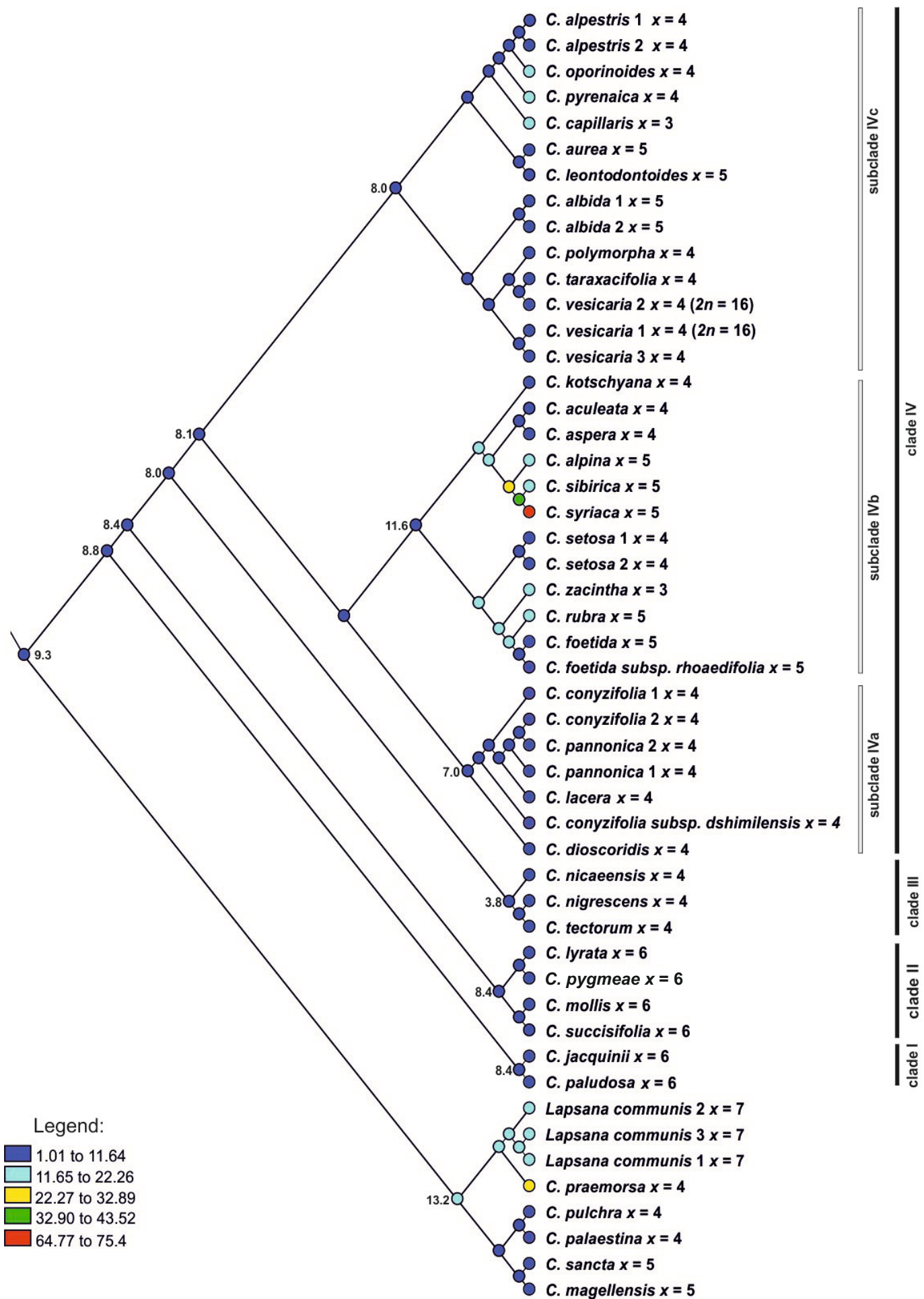

*Crepis sensus stricto*

*Lagosensis*

Supplement: Supplementary file 1 [file genes-12-01436-s001.zip › Senderowicz_et_al_Figure S6.pdf]

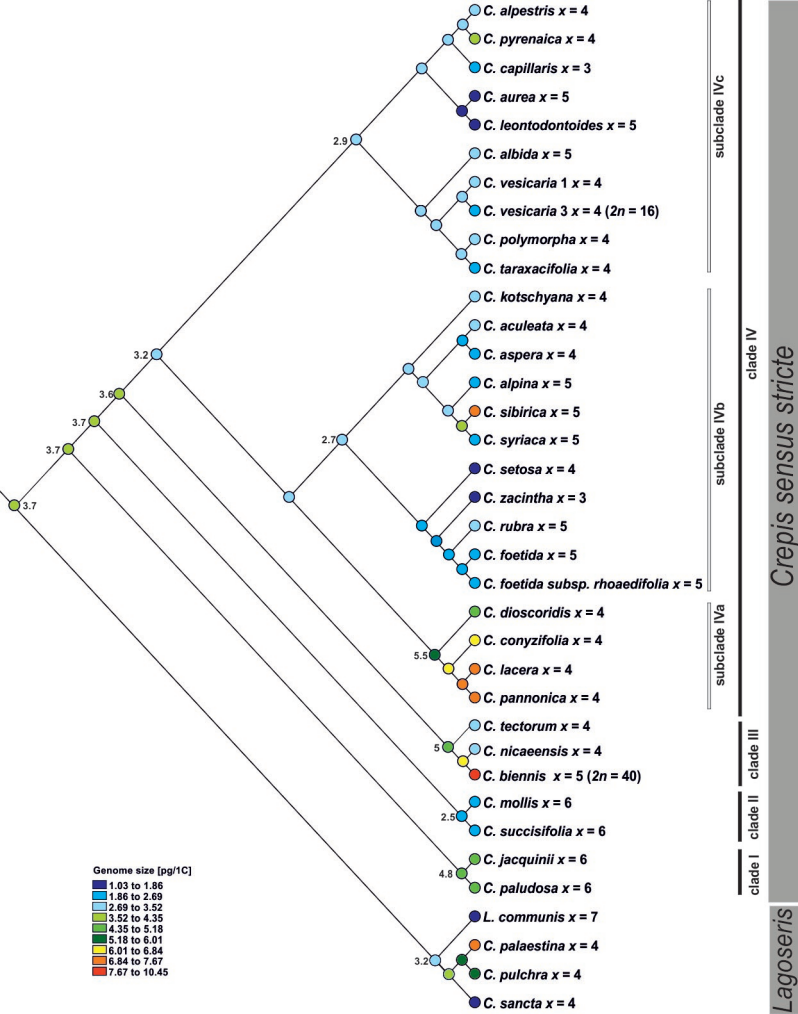

Supplement: Supplementary file 1 [file genes-12-01436-s001.zip › Senderowicz_et_al_Figure S7.pdf]

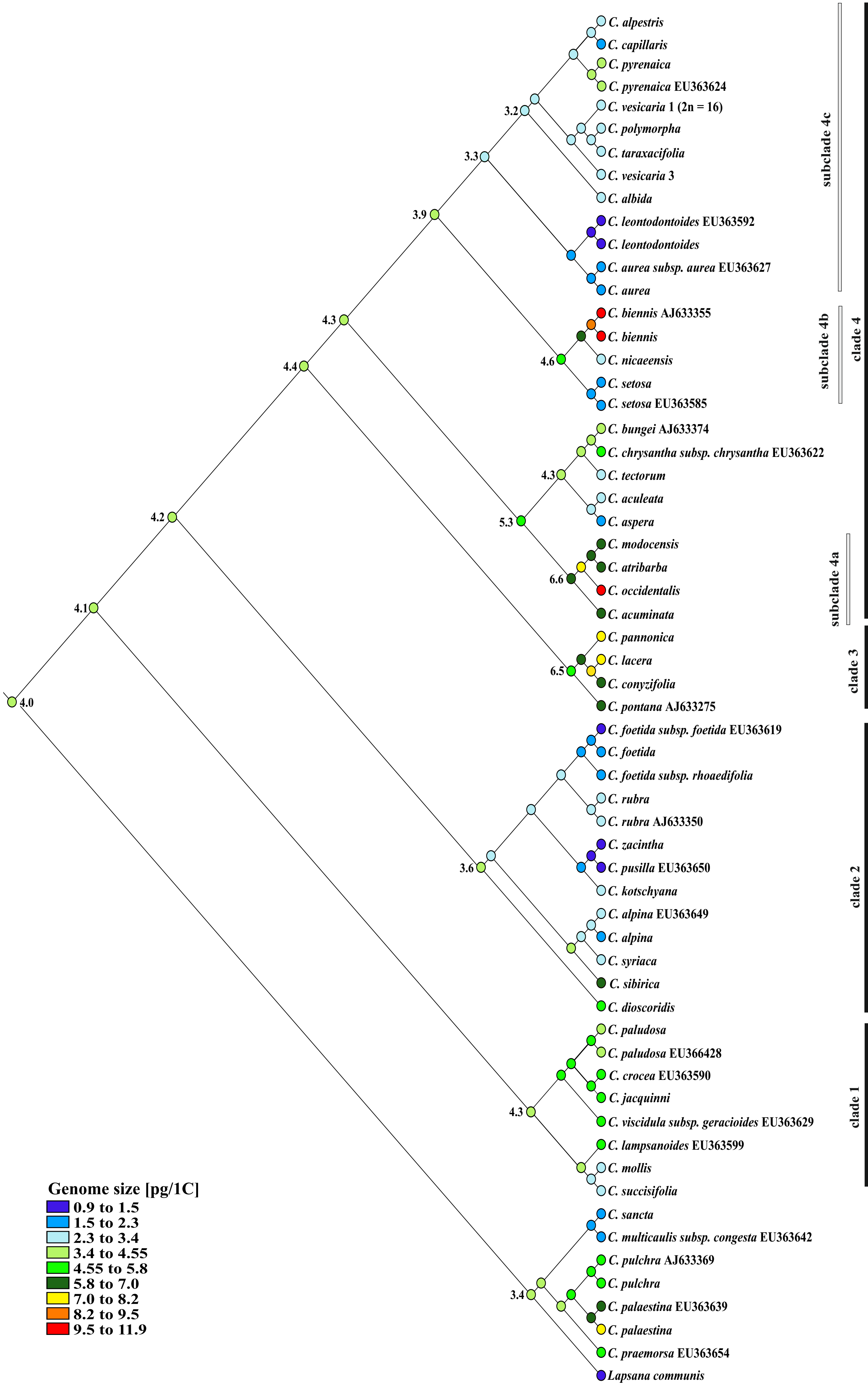

*Crepis sensu stricto*

*Lagoseris*

Supplement: Supplementary file 1 [file genes-12-01436-s001.zip › Senderowicz_et_al_Figure S8.pdf]

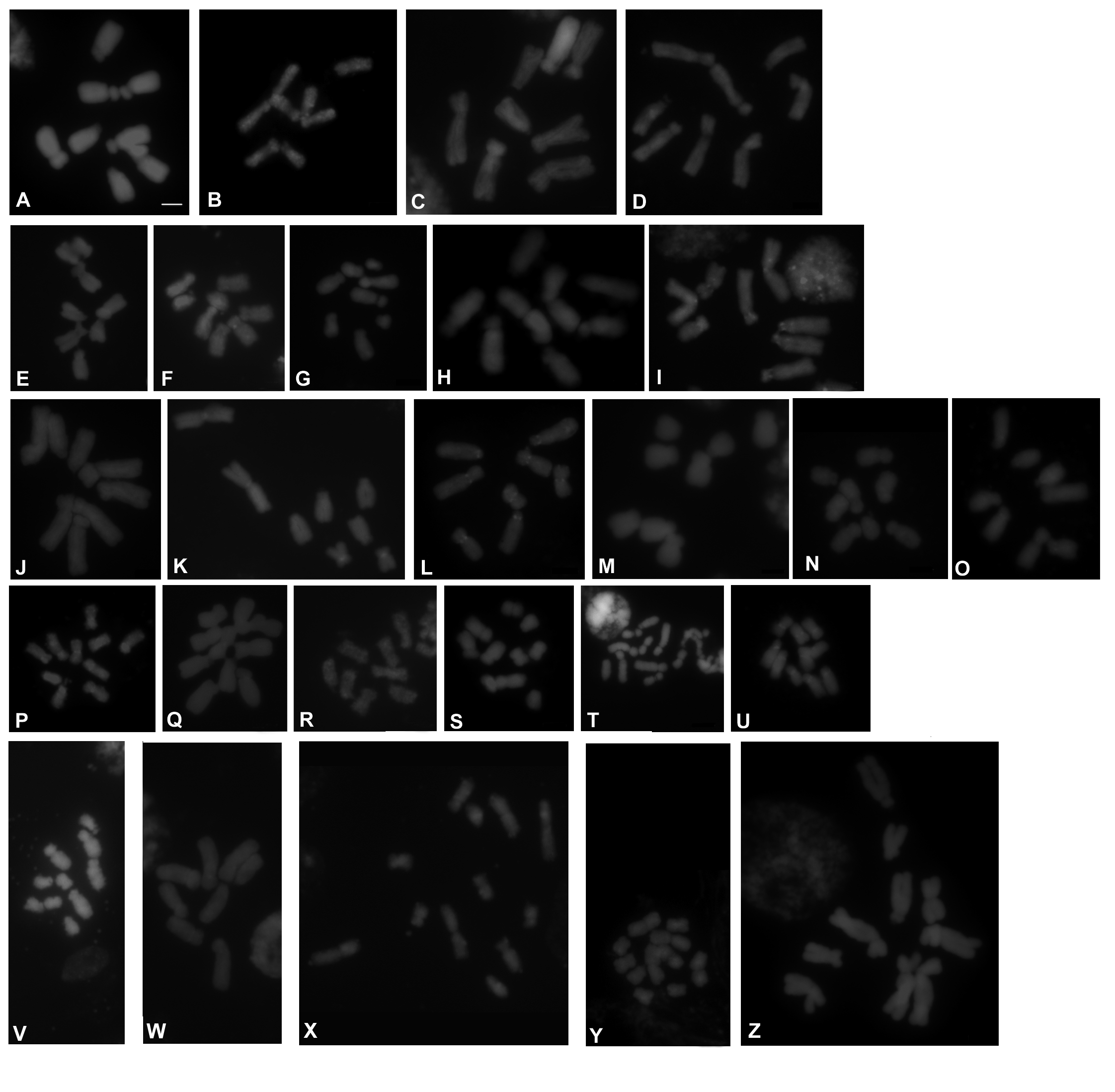

Supplement: Supplementary file 1 [file genes-12-01436-s001.zip › Senderowicz_et_al_Figure S9.tif]
