# Supplementary material for: Descending Dysploidy and Bidirectional Changes in Genome Size Accompanied Crepis (Asteraceae) Evolution
Source: Genes (Basel). 2021 Sep 17;12(9):1436. doi: 10.3390/genes12091436 (PMC8472258; doi:10.3390/genes12091436)
Supplement: Supplementary file 1 [file Senderowicz_et_al_Figure S1.pdf › Senderowicz_et_al_Figure S1.pdf]

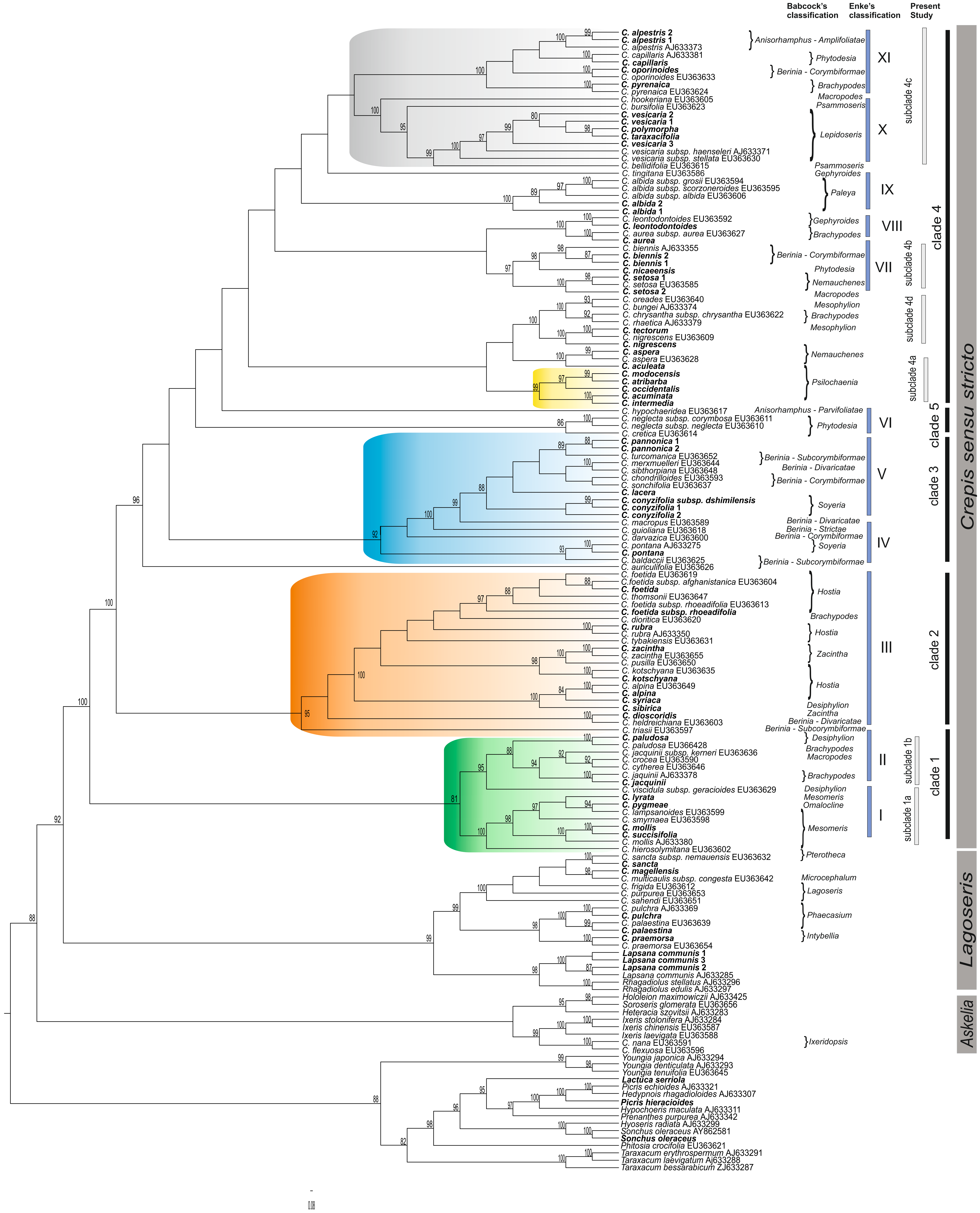
Babcock's classification
Enke's classification
Present Study
C. alpestris 2
C. alpestris 1
C. alpestris AJ633373
C. capillaris AJ633381
C. capillaris
C. oporinoides
C. oporinoides EU363633
C. pyrenaica
C. pyrenaica EU363624
C. hookeriana EU363605
C. bursifolia EU363623
C. vesicaria 2
C. vesicaria 1
C. polymorpha
C. taraxacifolia
C. vesicaria 3
C. vesicaria subsp. haenseleri AJ633371
C. vesicaria subsp. stellata EU363630
C. bellidifolia EU363615
C. tingitana EU363586
C. albida subsp. grosii EU363594
C. albida subsp. scorzoneroides EU363595
C. albida subsp. albida EU363606
C. albida 2
C. albida 1
C. leontodontoides EU363592
C. leontodontoides
C. aurea subsp. aurea EU363627
C. aurea
C. biennis AJ633355
C. biennis 2
C. biennis 1
C. nicaeensis
C. setosa 1
C. setosa EU363585
C. setosa 2
C. oreades EU363640
C. bungei AJ633374
C. chrysantha subsp. chrysantha EU363622
C. rhaetica AJ633379
C. tectorum
C. nigrescens EU363609
C. nigrescens
C. aspera
C. aspera EU363628
C. aculeata
C. modocensis
C. atribarba
C. occidentalis
C. acuminata
C. intermedia
C. hypochaeridea EU363617
C. neglecta subsp. corymbosa EU363611
C. neglecta subsp. neglecta EU363610
C. cretica EU363614
C. pannonica 1
C. pannonica 2
C. turcomanica EU363652
C. merxmuelleri EU363644
C. sibthorpiana EU363648
C. chondrilloides EU363593
C. sonchifolia EU363637
C. lacera
C. conyzifolia subsp. dshimilensis
C. conyzifolia 1
C. conyzifolia 2
C. macropus EU363589
C. guioliana EU363618
C. darvazica EU363600
C. pontana AJ633275
C. pontana
C. baldaccii EU363625
C. auriculifolia EU363626
C. foetida EU363619
C.foetida subsp. afghanistanica EU363604
C. foetida
C. thomsonii EU363647
C. foetida subsp. rhoeadifolia EU363613
C. foetida subsp. rhoeadifolia
C. dioritica EU363620
C. rubra
C. rubra AJ633350
C. tybakiensis EU363631
C. zacintha
C. zacintha EU363655
C. pusilla EU363650
C. kotschyana EU363635
C. kotschyana
C. alpina EU363649
C. alpina
C. syriaca
C. sibirica
C. dioscoridis
C. heldreichiana EU363603
C. triasii EU363597
C. paludosa
C. paludosa EU366428
C. jacquinii subsp. kerneri EU363636
C. crocea EU363590
C. cytherea EU363646
C. jaquinii AJ633378
C. jacquinii
C. viscidula subsp. geracioides EU363629
C. lyrata
C. pygmaea
C. lampsanoides EU363599
C. smyrnaea EU363598
C. mollis
C. succisifolia
C. mollis AJ633380
C. hierosolymitana EU363602
C. sancta subsp. nemauensis EU363632
C. sancta
C. magellensis
C. multicaulis subsp. congesta EU363642
C. frigida EU363612
C. purpurea EU363653
C. sahendi EU363651
C. pulchra AJ633369
C. pulchra
C. palaestina EU363639
C. palaestina
C. praemorsa
C. praemorsa EU363654
Lapsana communis 1
Lapsana communis 3
Lapsana communis 2
Lapsana communis AJ633285
Rhagadiolus stellatus AJ633296
Rhagadiolus edulis AJ633297
Hololeion maximowiczii AJ633425
Soroseris glomerata EU363656
Heteracia szovitsii AJ633283
Ixeris stolonifera AJ633284
Ixeris chinensis EU363587
Ixeris laevigata EU363588
C. nana EU363591
C. flexuosa EU363596
Youngia japonica AJ633294
Youngia denticulata AJ633293
Youngia tenuifolia EU363645
Lactuca serriola
Picris echioides AJ633321
Hedypnois rhagadioloides AJ633307
Picris hieracioides
Hypochoeris maculata AJ633311
Prenanthes purpurea AJ633342
Hyoseris radiata AJ633299
Sonchus oleraceus AY862581
Sonchus oleraceus
Phitosia crocifolia EU363621
Taraxacum erythrospermum AJ633291
Taraxacum laevigatum Aj633288
Taraxacum bessarabicum ZJ633287
Anisorhamphus - Amplifoliatae
Phytodesia
Berinia - Corymbiformae
Brachypodes
Macropodes
Psammoseris
Lepidoseris
Psammoseris
Gephyroides
Paleya
Gephyroides
Brachypodes
Berinia - Corymbiformae
Phytodesia
Nemauchenes
Macropodes
Mesophylion
Brachypodes
Mesophylion
Nemauchenes
Psilochaenia
Anisorhamphus - Parvifoliatae
Phytodesia
Berinia - Subcorymbiformae
Berinia - Divaricatae
Berinia - Corymbiformae
Soyeria
Berinia - Divaricatae
Berinia - Strictae
Berinia - Corymbiformae
Soyeria
Berinia - Subcorymbiformae
Hostia
Brachypodes
Hostia
Zacintha
Hostia
Desiphylion
Zacintha
Berinia - Divaricatae
Berinia - Subcorymbiformae
Desiphylion
Brachypodes
Macropodes
Brachypodes
Desiphylion
Mesomeris
Omalocline
Mesomeris
Pterotheca
Microcephalum
Lagoseris
Phaecasium
Intybellia
Ixeridopsis
XI
X
IX
VIII
VII
VI
V
IV
III
II
I
subclade 4c
subclade 4b
subclade 4d
subclade 4a
subclade 1b
subclade 1a
clade 4
clade 5
clade 3
clade 2
clade 1
Crepis sensu stricto
Lagoseris
Askelia
0.08
